# Selection of the Optimal Number of Topics for LDA Topic Model—Taking Patent Policy Analysis as an Example

**DOI:** 10.3390/e23101301

**Published:** 2021-10-03

**Authors:** Jingxian Gan, Yong Qi

**Affiliations:** School of Intellectual Property, Nanjing University of Science and Technology, Nanjing 210094, China; ganjx@njust.edu.cn

**Keywords:** LDA, topic model, optimal number of topics, patent policy

## Abstract

This study constructs a comprehensive index to effectively judge the optimal number of topics in the LDA topic model. Based on the requirements for selecting the number of topics, a comprehensive judgment index of perplexity, isolation, stability, and coincidence is constructed to select the number of topics. This method provides four advantages to selecting the optimal number of topics: (1) good predictive ability, (2) high isolation between topics, (3) no duplicate topics, and (4) repeatability. First, we use three general datasets to compare our proposed method with existing methods, and the results show that the optimal topic number selection method has better selection results. Then, we collected the patent policies of various provinces and cities in China (excluding Hong Kong, Macao, and Taiwan) as datasets. By using the optimal topic number selection method proposed in this study, we can classify patent policies well.

## 1. Introduction

From a text mining perspective, topics in a text corpus can be viewed as probability distributions of terms present in the corpus or clusters that define weights for those terms [1,2]. The LDA model (Latent Dirichlet Allocation) uses a probability distribution model to generate topics [3,4]. The principle is that a document is assumed to be generated from multiple topics according to a certain random probability distribution, and each topic is composed of words according to a random probability distribution. Thus, a document contains a collection of topics with different probabilities, and a topic contains a collection of words with different probabilities. Another important assumption of the LDA model is the “exchangeability” or “bag-of-words” hypothesis, which means that the importance of a word is not related to the order in which it appears in a document but rather to the frequency with which it appears [5,6]. This text processing approach of the LDA topic model makes the dimensionality of the represented text greatly reduced and has great advantages in the processing of massive text [7,8,9], which has led to the widespread use of LDA topic generation models in knowledge mining, intelligence analysis, and other fields in recent years [5,10,11,12].

However, the LDA topic model has a problem that it does not give the optimal number of topics for the text itself, and the exact number of topics needs to be determined by the model user in other ways [13]. The determination of the number of topics is crucial for text analysis, but how to determine the optimal number of topics for the LDA topic model has not been well studied. There are few studies about how many words should be selected to represent under each topic [14,15]. The commonly used perplexity metric also has some drawbacks [16]. In order to better select the optimal number of topics for LDA models, this study proposes a comprehensive judgment index based on perplexity, isolation, stability, and coincidence.

## 2. Literature Review

Some studies have shown that the effectiveness of topic classification in LDA models is closely related to the selection of the number of topics. If the number of topics selected is too small, the meaning under each topic will be too broad; if the number of topics selected is too large, it will lead to an over-clustering of data, producing useless topics or topics with too much similarity. To address this issue, Blei et al. (2003) proposed a perplexity model to determine the quality of topic models with different numbers of topics [3]. They compared the output results by calculating the perplexity of the topic models with different number of topics, and the smaller the perplexity, the better the model with that number of topics. Although the perplexity can judge the predictive ability of the topic training model to a certain extent, when the number of topics is selected by the perplexity, the number of topics selected is often large, and similar topics are likely to appear, resulting in a low recognition of the topics. Arun (2010) uses the JS (Kullback–Leibler) scatter to compare the similarity between topics and select the optimal number of topics, and the JS scatter is smaller when the number of topics is close to the optimal value [17]. He et al. (2015) set different genetic degrees for each topic based on topic strength, use JS (Jensen–Shannon) scatter to calculate the association between topics and screen out similar topics [18]. Both JS divergence and JS divergence methods are based on the principle that the similarity between topics separated by a good topic model should be as small as possible. The method adopted by Guan et al. (2016) uses a combination of perplexity and JS divergence to select the optimal number of topics, which combines the advantages of JS divergence and improves the problem of using the perplexity formula alone, which leads to the selection of too large a number of topics [7]. The coherence method proposed by Röder et al. (2015) evaluates the classification effectiveness of a topic by measuring the degree of semantic co-occurrence between high-probability words in the topic [19]. Greene et al. (2014) verified the topic stability of the Non-negative Matrix Factorization (NMF) model based on the similarity of the topic word lists in the training and test sets, by which a more robust number of topics can be obtained [1]. However, this method may have the problem that the topics of multiple training sets point to the same test topic.

Then, we use a toy example to show the differences and problems of the most commonly used methods of perplexity, JS divergence, and coherence. Assume that the following four documents are available for LDA training:

Doc 1 = [‘apple’, ‘pear’, ‘pear’, ‘apple’, ‘dog’]
Doc 2 = [‘cat’, ‘dog’, ‘pig’, ‘cat’, ‘dog’]
Doc 3 = [‘apple’, ‘pear’, ‘apple’, ‘dog’]
Doc 4 = [‘pig’, ‘dog’, ‘dog’].

The two test set documents are:

Test1 = [‘pear’, ‘apple’, ‘pig’]
Test2 = [‘cat’, ‘dog’].

First, the LDA model is used to train four documents with the number of topics *k* set from two to four, and the “topic-word” probabilities are calculated for different numbers of topics, as shown in Table 1.

With human intuition, the training set can be divided into two topics, animals {dog, cat, pig} and fruits {apple, pear}. We use the top two words in the probability ranking to determine the content of the topic. As can be seen from Table 1, when the number of topics is two, the first two words in Topic1 are ‘apple’ and ‘pear’, and the content of the topic can be judged to be fruit. The first two words in Topic2 are ‘dog’ and ‘cat’, so we can decide that the topic is about animals.

When the number of topics is three, the first two words in Topic1 are “apple” and “pear”, which can be judged as a fruit topic. The first two words in Topic2 are “dog” and “cat”, which can be judged as an animals topic. The first two words in Topic3 are also animal-related, “dog” and “pig”.

When the number of topics is four, the first two words in Topic1 are ‘apple’ and ‘dog’, and the content of the topic cannot be determined. The first two words in Topic2 are animal-related, ‘dog’ and ‘cat’, which can be judged as an animals topic. The first two words in Topic3 are ‘apple’ and ‘dog’, and the content of the topic cannot be determined. The first two words in Topic4 are fruit-related, ‘apple’ and ‘pear’, which can be judged as a fruit topic.

It can be seen that the LDA model with two topics is the most consistent with human intuition. In contrast, the LDA model with three topics has two animal-related repetitive topics, and the LDA model with four topics has two unknown topics that cannot be distinguished.

Second, the topic probabilities of the test set documents are calculated for the LDA model with different numbers of topics. Table 2 shows the “document-topic” probabilities of the test set calculated by the LDA model for different numbers of topics.

Combining the “topic-word” probability in Table 1 and the “document-topic” probability in Table 2, we can determine the topic content of the test document. When the number of topics is two, the probability that the content of Test1 belongs to Topic1 Fruit is 0.6808 and the probability that it belongs to Topic2 Animals is 0.3192. The probability that the content of Test2 belongs to Topic1 Fruit is 0.1821 and the probability that it belongs to Topic2 Animals is 0.8179.

When the number of topics is three, the probability that the content of Test1 belongs to Topic1 Fruit is 0.5932, the probability that it belongs to Topic2 Animals is 0.3182, and the probability that it belongs to Topic3 Animals is 0.0885. The probability that the content of Test2 belongs to Topic1 Fruit is 0.1151, the probability that it belongs to Topic2 Animals is 0.7711, and the probability that it belongs to Topic3 Animals is 0.1138.

When the number of topics is four, the probability that the content of Test1 belongs to Topic1 Unknown topic is 0.0655, the probability that it belongs to Topic2 Animals is 0.3075, the probability that it belongs to Topic3 Unknown topic is 0.0632, and the probability that it belongs to Topic4 Fruit is 0.5637. The probability that the content of Test2 belongs to Topic1 Unknown topic is 0.0842, the probability of belonging to Topic2 Animals is 0.7476, the probability of belonging to Topic3 Unknown topic is 0.0837, and the probability of belonging to Topic4 Fruit topic is 0.0845.

It is also clear from the topic determination of the test set documents by the LDA models with different numbers of topics that the “document-topic” probability obtained by the model with two topics is most consistent with our intuitive judgment. The LDA models with three and four topics produce duplicate and unknown topics, which interfere with the topic probability of the test documents and affect our judgment of the topic content of the documents.

From the above analysis, it can be seen that the LDA model with two topics is most consistent with our intuitive judgment. Since this toy example is too simple, it is not difficult to manually select the optimal number of topics, but for a large number of documents, it will be very difficult to judge manually. Next, we will use different methods to select topics for this toy example to see whether the existing methods can select the optimal LDA model.

Third, the number of topics for the LDA model was selected using the perplexity, JS divergence, coherence, and stability methods as well as our proposed optimal topic number selection method. Since the toy example has fewer words and texts, with only two words related to fruits and three words related to animals, the coherence, stability method, and our proposed optimal topic number selection method were calculated by using the top two words ranked for each topic to indicate the topic meaning. The calculation results are shown in Table 3.

The perplexity method considers the predictive ability of the model for documents. The principle is that if most of the words in a document are the top words in the probability ranking of the topics generated by LDA, then the prediction of the topic to which the document belongs is more accurate. On the contrary, it is difficult to determine the topic of the document. Therefore, the lower the perplexity, the better the prediction ability of the model. From Table 3, it can be seen that the optimal number of topics selected by the perplexity method is four.

The JS divergence method considers that the topics generated by a topic model should be well differentiated from each other, and JS divergence is a measure of the difference in word-probability distributions between topics to determine whether a topic model is good or bad. Therefore, for JS divergence, the greater the difference in the distribution of “topic-word” probabilities among topics, the better the topic generation model. Table 3 shows that the optimal number of topics selected by the JS divergence method is four.

The coherence method considers the coherence of semantic information in the model, that is, the conditional likelihood is estimated for the combination of words for which the document contains the first n of the topic words at the same time. Therefore, for the coherence method, the higher the probability that the words with the highest topic ranking can appear in a document at the same time, the better the classification effect of the model. From Table 3, we can see that the optimal number of topics selected by the coherence method is 2.

The optimal number of topics selection method we propose adds the judgment of model stability and coincidence based on the consideration of the predictive ability and topic isolation of the model, as shown in Table 3 for stability and coincidence. The specific calculation method will be introduced in Section 3. From Table 3, we can see that the optimal number of topics selected by our proposed method is two.

This toy example shows that both the perplexity method and the JS divergence method select the number of topics to be four, which is the maximum number of topics for our training of this small sample. The selection results of these two methods are not consistent with our intuitive judgment. From Table 2, we can see that the LDA model with four topics has unknown topics whose contents we cannot determine. The optimal number of topics selected by both the coherence method and our proposed method is two, which is consistent with our intuitive judgment of the number of topics. However, the sample of this toy example is too small to cover the various situations that may be faced in realistic data processing. In Section 3, we will give further examples to analyze the problems that realistic model training may face and how the stability and coincidence indexes can be solved. In Section 4, we further compare the effectiveness of these existing methods with our proposed method for topic number selection by using the 20news_groups dataset, which has larger data compared to this toy example.

By analyzing the existing studies, it can be seen that although the LDA model can generate topics well by the probability distribution of words, it cannot give an optimal judgment about the number of topics to be selected. Most of the existing studies perform topic number selection from a certain feature that a good topic classification model should have. For example, the perplexity method considers that a good topic classification model should be able to effectively classify unknown documents outside the training set. The JS divergence or JS divergence approach considers that the less similar the content among the topics classified, the better the topic classification model. Both the coherence method and the stability method proposed by Greene judge the goodness of the topic classification by the similarity of the words within the same topic in the model. Although these existing topic selection methods are not comprehensive for topic classification, they also provide a good basis for us to think about how to judge whether a topic classification model is good or not.

Based on the existing studies, it is known that a better topic selection model should have the following characteristics: (1) The topic model has certain predictive ability; i.e., the model can classify the unknown input corpus well. (2) There should be a certain degree of isolation between different topics generated by the model; i.e., the occurrence of duplicate topics is avoided. (3) The words within each topic generated by the topic model are correct. (4) The generated model is robust; i.e., the data from the same source consistently generated similar solutions. Based on these characteristics, this study proposes a comprehensive method for determining the optimal number of LDA topics. There is no research to select the number of topics for LDA models from a comprehensive perspective, and most of the existing methods are one-time tests, which make it difficult to ensure that the models can be reliably and stably implemented repeatedly. In view of the limitations of existing studies, this paper will propose a comprehensive method for judging the optimal LDA topic number based on the above four characteristics that a good topic model should have.

## 3. LDA Topic Model and Optimal Topic Number Selection Method

### 3.1. Latent Dirichlet Allocation (LDA)

**Figure 1:**
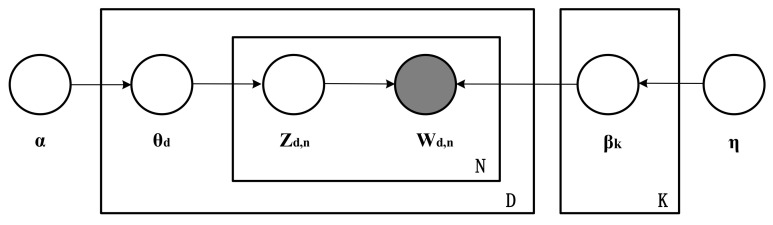
Schematic diagram of the LDA model.

Latent Dirichlet Allocation (LDA) is a document topic generation model proposed by Blei et al. (2003) after introducing the Dirichlet distribution based on Probabilistic Latent Semantic Analysis (PLSA). LDA is a classical model for generating Bayesian probability models, which is based on the assumption that documents are generated from multiple topics according to a certain random probability distribution, and each topic is composed of words according to a random probability distribution. Thus, a document is a collection of topics with different probabilities, and a topic is a collection of words with different probabilities. The LDA topic model calculates the probability of the conditional distribution (posterior distribution) of the hidden variables given the observed variables by using the joint distribution. The observed variable is the set of words and the hidden variable is the topic, whose probability map model is shown in Figure 1.

The joint distribution of the observed and hidden variables corresponding to the generation process of the LDA model is shown in Equation (1).

(1) $$p\left(\beta_{1:K},\;\theta_{1:D},z_{1:D},w_{1:D}\right)=\prod_{i=1}^{K}p\left(\beta \right)\prod_{d=1}^{D}p\left(\theta_{d}\right)\left(\prod_{n=1}^{N}p\left(z_{d,n}|\theta_{d}\right)p\left(w_{d,n}|\beta_{1:K},z_{1:D}\right)\right)$$

The concatenated symbol in Equation (1) describes the dependence of the random variables. Here, β denotes the topic, θ denotes the probability of the topic, z denotes the topic of a particular document or word, and w is the word. $\beta_{1:K}$ is the set of all topics, where $\beta_{k}$ is the distribution of the words of the *k*th topic. The proportion of that topic in the *d*th document is $\theta_{d}$, where $\theta_{d,k}$ denotes the proportion of the *k*th topic in the *d*th document. The set of topics contained in the *d*th document is $z_{d}$, where $z_{d,n}$ is the topic to which the *n*th word in the *d*th document belongs. The set of all words in the *d*th document is denoted $w_{d}$, where $w_{d,n}$ is the *n*th word in the *d*th document. $p\left(\beta \right)$ denotes a specific topic selected from the set of topics, $p\left(\theta_{d}\right)$ denotes the probability of that topic in a specific document, $\prod_{n=1}^{N}p\left(z_{d,n}|\theta_{d}\right)$ denotes the probability that the *n*th word of the document corresponds to the topic to which it belongs when the topic is determined, and $p\left(w_{d,n}|\beta_{1:K},z_{1:D}\right)$ denotes the joint probability of the topic to which the *n*th word of the document belongs and the word.

### 3.2. Optimal Topic Number Selection Method

#### 3.2.1. Perplexity

Perplexity is calculated as follows.

(2) $$perplexity\left(D_{test}\right)=\exp\{\frac{-\sum_{d=1}^{M}\log p\left(w_{d}\right)}{\sum_{d=1}^{M}N_{d}}\}$$

where *M* is the size of the test corpus, $N_{d}$ is the size of the *d*th text (that is, the number of words), and $p\left(w_{d}\right)$ denotes the probability that word $w_{d}$ is generated in the document.

Existing studies found that although the perplexity can judge the predictive ability of the topic training model to a certain extent, when the number of topics is selected by the perplexity, the number of topics selected is often too large and does not converge, and similar topics are likely to appear, resulting in a low discrimination of topics. Therefore, this study further judges the number of topics by the inter-topic similarity of the training model to improve the accuracy.

#### 3.2.2. Isolation

The isolation of the topic model is usually calculated using either the Kullback–Leibler divergence (JS divergence) or the Jensen–Shannon divergence (JS divergence). Since the JS divergence does not satisfy the symmetry and triangular inequality [17], the JS divergence is used for the calculation in this research section.

The JS divergence formula is based on the deformation of the JS divergence formula. JS divergence is also called relative entropy; for two probability distributions *P* and *Q*, the more similar they are, the smaller the JS divergence, and the more isolated they are, the larger the JS divergence value. JS divergence is calculated by the following formula.

(3) $$\mathrm{KL}(P||Q)=-\sum_{x\in X}P\left(x\right)log\frac{1}{P\left(x\right)}+\sum_{x\in X}p\left(x\right)log\frac{1}{Q\left(x\right)}$$

Since the JS divergence formula is asymmetric, swapping the positions of *P* and *Q* will give different results, so the JS scattering formula after adjusting to symmetry is as follows.

(4) $$\mathrm{JS}(P||Q)=\frac{1}{2}\mathrm{KL}\left(P||\frac{P+Q}{2}\right)+\frac{1}{2}\mathrm{KL}\left(Q||\frac{P+Q}{2}\right)$$

The JS divergence takes a value between 0 and 1. When the distributions of *P* and *Q* are the same, the JS divergence takes a value of 0.

In the calculation, first, the data are divided into training and test sets, and the training set text is used to complete the training of the LDA model under the given number of topics *k*. Then, the test set text is used as the input of the model to get the word probability distribution of the test set corresponding to *k* topics, and the average probability of each word is calculated based on the probability distribution of words corresponding to different topics. Finally, the standard deviation of the JS distance between the word probability distribution of each topic and the average probability distribution is calculated as the isolation degree when the number of topics is k. The calculation formula is as follows.

(5) $$\sigma_{\mathrm{JS}}=\sqrt{\frac{\sum_{i=1}^{k}{\left[\mathrm{JS}(S_{i}||S_{ave})\right]}^{2}}{k}}$$

where $S_{i}$ denotes the word distribution of the *i*th topic and $S_{ave}$ denotes the average word distribution of the *k* topics.

#### 3.2.3. Stability

The stability of a clustering algorithm is expressed as the ability of this algorithm to consistently produce similar solutions for data originating from the same source [20]. Greene et al. (2014) proposed a method to verify the topic stability of the Non-negative Matrix Factorization (NMF) model using the similarity of the list of topic words, by which a more robust topic count can be obtained [1]. Drawing on this approach, this study improves it as part of the optimal topic number determination for LDA.

##### Topic Word Ranking Similarity Measurement

Topic model algorithms usually take the ranking of words under each topic as their output. In the LDA model, these words are ranked based on the probability distribution of the words under the topic. Assume that the two topic lists are $R_{i}$ and $R_{j}$, and each topic contains a word ranking list. The ranking of words in the LDA model reflects the importance of words to this topic, and each topic is given a probabilistic ranking of all words in the entire corpus dictionary. Then, the similarity between a pair of ranked lists $\left(R_{i},R_{j}\right)$ can be evaluated using the symmetry metric. Most similarity algorithms do not take into account the ranking position of the words, and if the similarity calculation is performed for all words in each topic, the resulting similarity value is 1. Therefore, this study will represent each topic by the first *t* words of that topic, where *t* is less than the total number of words. This substitution not only makes the similarity between topics comparable but also greatly reduces the computational effort. In this study, the Jaccard index was used to calculate the similarity of word ranking lists for each pair of topics. An average Jaccard index (AJ index) was defined to calculate the word ranking, since it has a large impact on the meaning of the topic. The AJ index calculates the average of the word similarity for each pair of topics ranked from 1 to *t*. The calculation is as follows.

(6) $$\mathrm{AJ}\left(R_{i},R_{j}\right)=\frac{1}{t}\sum_{d=1}^{t}\gamma_{d}\left(R_{i},R_{j}\right)$$

(7) $$\gamma_{d}\left(R_{i},R_{j}\right)=\frac{R_{i,d}\cap R_{j,d}}{R_{i,d}\cup R_{j,d}}\;$$

In the above equation, $R_{i,d}$ denotes the list of the top *d* words of the topic at the selected vocabulary depth of *t*. The AJ index calculation is a symmetric calculation method that reflects both the degree of vocabulary relatedness of the two topics and the similarity of the ranking of the words.

As an example, Table 4 shows the list of the top *d* words for two topics $R_{i}$ and $R_{j}$ at a word depth of *t* equal to 5.

As shown in Table 4, the values of $Jac_{d}$ represent the similarity of topics with the same number of words only, while the values of AJ represent the distribution of words in addition to the similarity of word lists in topics.

##### Stability of the Topic Model

Assume that two different sets containing *k* topics are $S_{x}=\left(R_{x1},\ldots \ldots ,R_{xk}\right)$ and $S_{y}=\left(R_{y1},\ldots \ldots ,R_{yk}\right)$. Construct a similarity matrix M of k×k. Each $M_{ij}$ value represents the AJ similarity of a pair of topic word ranking lists $R_{xi}$ and $R_{xj}$ in two sets. Then, the similarity matrix M is used to find the $R_{yi}$ with the highest AJ similarity to it for each $R_{xj}$ in the set $S_{x}$. Finally, the AJ similarity of the selected *k* optimal consistent combinations is summed and averaged, which is the stability of the subject set $S_{x}$ and $S_{y}$. The formula is as follows.

(8) $$stability\left(S_{x},S_{y}\right)=\frac{1}{k}\sum_{i=1}^{k}\mathrm{AJ}\left(R_{xi},\pi \left(R_{xi}\right)\right)$$

The $\pi \left(R_{xi}\right)$ in the above equation denotes the most matching arrangement with $R_{xi}$ in the $S_{y}$ set. The coherence of $S_{x}$ and $S_{y}$ takes a value between 0 and 1. The more coherent two topic sets are, the greater the value of their coherence, which takes the value of 1 when the two topics are the same.

An example is given below. Suppose there are two topic sets S1 and S2 that both contain three topics.

The set of topics for s1 is:

$$R_{11}=\left\{Technology,\;Innovation,\;Patents\right\}$$

$$R_{12}=\left\{Infringement,\;Protection,\;Enforcement\right\}$$

$$R_{13}=\left\{Economy,\;Financing,\;Pledge\right\}.$$

The set of topics for s2 is:

$$R_{21}=\left\{Protection,\;Infringement,\;Counterfeiting\right\}$$

$$R_{22}=\left\{Economy,\;pledge,\;investment\right\}$$

$$R_{23}=\left\{Invention,\;Technology,\;Innovation\right\}$$

Then, the $S_{1}$ and $S_{2}$ similarity matrix M is:

$$M=\begin{array}{cccc} & R_{21} & R_{22} & R_{23}\\ R_{11} & 0.00 & 0.00 & 0.28\\ R_{12} & 0.5 & 0.00 & 0.00\\ R_{13} & 0.00 & 0.61 & 0.00 \end{array}.$$

The optimal coherence combination is:

$$\pi =\left(R_{11},R_{23}\right),\left(R_{12},R_{21}\right),\left(R_{13},R_{22}\right).$$

The stability of $S_{1}$ and $S_{2}$ is:

$$stability\left(S_{1},S_{2}\right)=\frac{0.28+0.5+0.61}{3}=0.46.$$

#### 3.2.4. Coincidence

Three situations may occur when using the training model with the test model for stability measurement: (1) In the coherence combination of the training model and the test model, there are no repeatedly selected topics in the test model, as follows.

$$\begin{array}{cccc} & R_{21} & R_{22} & R_{23}\\ R_{11} & 0.00 & 0.00 & 0.28\\ R_{12} & 0.5 & 0.00 & 0.00\\ R_{13} & 0.00 & 0.61 & 0.00 \end{array}$$

(2) In the coherence combination of the training model and the test model, there are repeatedly selected topics in the test model, and there are some topics that are not selected, as follows.

$$\begin{array}{cccc} & R_{21} & R_{22} & R_{23}\\ R_{11} & 0.28 & 0.00 & 0.00\\ R_{12} & 0.5 & 0.00 & 0.00\\ R_{13} & 0.00 & 0.61 & 0.00 \end{array}$$

(3) In the coherence combination of training and testing models, there exists a certain training topic that appears to have the same maximum AJ value as the topics of more than two testing models, as follows.

$$\begin{array}{cccc} & R_{21} & R_{22} & R_{23}\\ R_{11} & 0.00 & 0.5 & 0.5\\ R_{12} & 0.5 & 0.00 & 0.00\\ R_{13} & 0.00 & 0.61 & 0.00 \end{array}$$

An optimal topic classification should be such that every topic word sequence under the training model corresponds to every topic word sequence under the test model. There are two cases that result in the topics of the training model and the test model not corresponding exactly. The first is that since the sampled dataset is smaller than the overall dataset, in some sampling, it may result in the topics trained in the training set not corresponding exactly to the test model. The second one is that when the number of topics in the test model is too large, it is easy to generate similar duplicate topics, which leads to the same value when calculating the stability of the training and test models. However, this aspect is mostly lacked in the existing studies. In order to improve the accuracy of the model stability measurement and make its applicability more complete, in this study, we add a consideration of whether the training model and the test model completely coincide in terms of topic connotation to the model stability measurement.

The combination of the maximum coherence of the sets $S_{x}$ and $S_{y}$ can be derived according to the topic model similarity measure in Section 3.2.3. The number of times each topic in the test model overlaps with the topic of the training model is counted and accumulated, and finally, it is divided by the number of coincident topics to obtain the coincidence of the test and training models under that number of topics. The calculation formula is as follows.

(9) $$Coincidence=\frac{1}{c}\sum_{i=1}^{k}count\left(\pi \left(R_{xi}\right)\right)$$

In the above equation, *c* denotes the number of topics in the test model and the training model that coincide, and *k* denotes the number of topics.

As an example, let two sets of topics that both contain three topics, and the model similarity measures of training model $S_{1}=\left\{R_{11},\;R_{12},R_{13}\right\}$ and test model $S_{2}=\left\{R_{21},R_{22},R_{23}\right\}$ be as follows. In the first case, there are no duplicate selected topics in the test model for the coherent combination of the training and test models.

$$\begin{array}{cccc} & R_{21} & R_{22} & R_{23}\\ R_{11} & 0.00 & 0.00 & 0.28\\ R_{12} & 0.5 & 0.00 & 0.07\\ R_{13} & 0.00 & 0.61 & 0.00 \end{array}$$

The optimal coherence combination is,

$$\pi =\left(R_{11},R_{21}\right),\left(R_{12},R_{21}\right),\left(R_{13},R_{22}\right).$$

$R_{21}$, $R_{22}$ and $R_{23}$ in the test model occur once each; thus,

$$Coincidence=\frac{1}{3}\left(1+1+1\right)=1.$$

In the second case, in the coherent combination of the training model and the test model, there are duplicate selected topics in the test model, and there are some topics that are not selected.

$$\begin{array}{cccc} & R_{21} & R_{22} & R_{23}\\ R_{11} & 0.28 & 0.00 & 0.00\\ R_{12} & 0.5 & 0.00 & 0.00\\ R_{13} & 0.00 & 0.61 & 0.00 \end{array}$$

The optimal coherence combination is

$$\pi =\left(R_{11},R_{21}\right),\left(R_{12},R_{21}\right),\left(R_{13},R_{22}\right).$$

The test model has two occurrences of $R_{21}$, zero occurrences of $R_{22}$, and one occurrence of $R_{23}$. Thus,

$$Coincidence=\frac{1}{2}\left(2+0+1\right)=1.5.$$

In the third case, there is a coherent combination of training and test models in which a certain training topic has the same maximum AJ value as more than two test model topics.

$$\begin{array}{cccc} & R_{21} & R_{22} & R_{23}\\ R_{11} & 0.00 & 0.5 & 0.5\\ R_{12} & 0.5 & 0.00 & 0.00\\ R_{13} & 0.00 & 0.61 & 0.00 \end{array}$$

The optimal coherence combination is

$$\pi =\left(R_{11},R_{22}\right),\left(R_{11},R_{23}\right),\left(R_{12},R_{21}\right),\left(R_{13},R_{22}\right).$$

The test model has one occurrence of $R_{21}$, two occurrences of $R_{22}$, and one occurrence of $R_{23}$. Thus,

$$Coincidence=\frac{1}{3}\left(1+2+1\right)=1.33.$$

The value of model coincidence is greater than or equal to 1. When the value of model coincidence is 1, it means that the subjects of the training model and the test model can completely coincide, and the larger the value of coincidence means that the coincidence of the training model and the test model is worse. As in the case of model stability measurement, this study obtains multiple model coincidence values through multiple sampling training and finally averages them to obtain the average coincidence value as a basis for judgment.

#### 3.2.5. Optimal Topic Number Selection

A reliable topic model should have three characteristics: first, the model has a good predictive capability, which means the model has low perplexity; second, there is a certain degree of isolation between different topics to avoid the appearance of duplicate topics, which means the similarity between topics is low; third, the model has a high stability, which means the model is repeatable. Fourth, the directionality of the topics of the model is unique and reliable, which means that the model has a low degree of coincidence.

Existing studies have found that selecting the number of topics by the perplexity value often leads to the selection of too large a number of topics. In this study, the perplexity of the topic model is combined with the model isolation, stability, and the coincidence index proposed in this study to obtain a better number of topics by cyclically training and testing the model. It is important to note that here, since usually, the calculated values of the perplexity method are much larger than several other metrics. In order to avoid the influence bias among the indicators, we take the logarithm of the perplexity when using our proposed method. The final topic superiority scores are defined as follows.

(10) $$Topic\_score=\frac{Perplexity\times Coincidence}{\sigma_{\mathrm{JS}}\times Stability}$$

**Figure 2:**
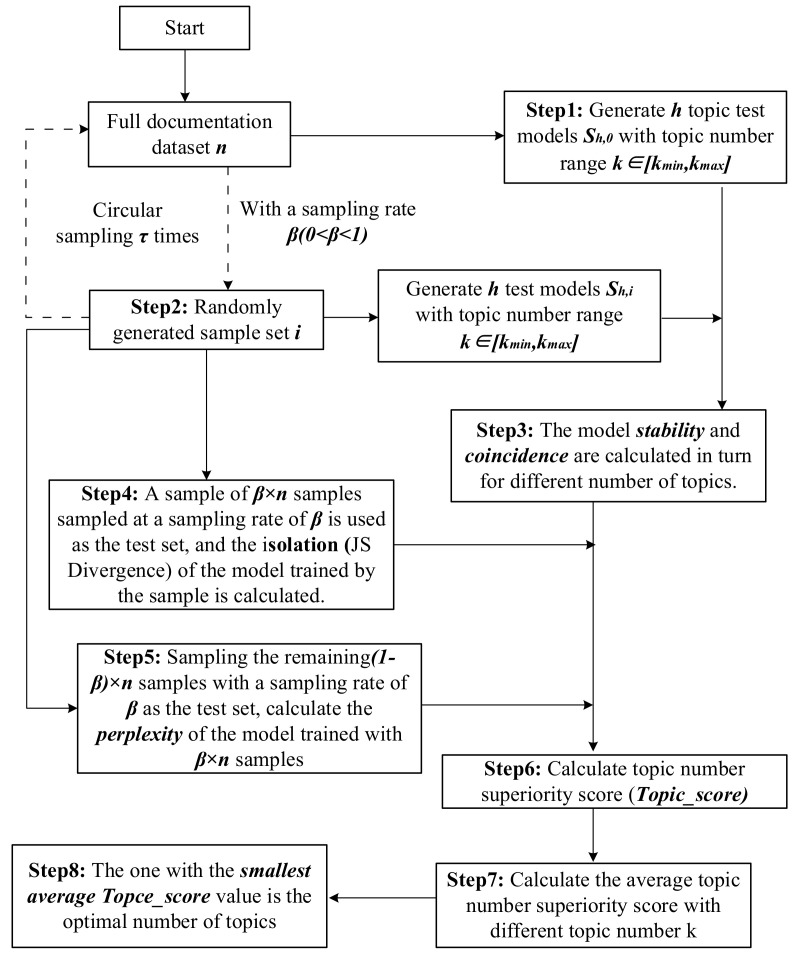
Steps for calculating the average topic superiority score.

To calculate the degree of model stability for different topic numbers k, first, an initial topic model is generated based on the complete dataset, and this model for the complete data is also the test model for the subsequent calculation of topic consistency, which is defined as $S_{0}$. Second, τ samples are randomly selected from the overall dataset n with a sampling rate of β(0<β<1). Third, a model is generated based on these τ samples as well, which is defined as $\left\{S_{1},\ldots \ldots ,S_{\tau }\right\}$. Fourth, the stability and coincidence of each random sample topic model with the test model are calculated. In addition, the remaining $\left(1-\beta \right)*n$ samples after the test samples are taken as the training set for the test samples, and the perplexity and isolation of each test sample are calculated. Finally, the *k* topic superiority scores of the τ sample models are averaged, and the minimum number of topics with the average topic superiority score value is the optimal number of topics *k*. The calculation steps are shown in Figure 2.

The calculation steps are as follows:

Step 1: The complete document set n (after data cleaning) is trained by the LDA model to generate topic test models with different numbers of topics $S_{h,0}$.

Step 2: Samples are drawn from the complete document set n with a sampling rate of β as the test set. Then, the test set is used to train to generate test model $S_{h,i}$ under different numbers of topics.

Step 3: Calculate the stability of the test model $S_{h,0}$ and the test model $S_{h,i}$ for the same number of topics. Calculate the coincidence of the test model $S_{h,0}$ and the test model $S_{h,i}$ for the same number of topics.

Step 4: Calculate the isolation of models under different numbers of topics by using the β×n documents in the test set obtained from each sampling.

Step 5: Calculate the perplexity of models under different numbers of topics by using the remaining $\left(1-\beta \right)\times n$ documents obtained from each test set sampling.

Step 6: Calculate the topic superiority index under different numbers of topics by using the stability, coincidence, isolation, and perplexity obtained from Steps 3 to 5.

Step 7: Repeat Steps 2 to 6 to obtain the topic superiority index of the τ test and test models, and calculate the average topic superiority index under different numbers of topics.

Step 8: The number of topics corresponding to the smallest average topic superiority index is taken as the optimal number of topics.

## 4. Experimental Analysis and Comparison

### 4.1. Data Source

This section uses three datasets, 20news_groups [21], WOS_46985 [22], and AG_news [23], to compare and validate our proposed topic number selection method. The original 20news_groups dataset contains a total of 18,846 texts on 20 topics, and these texts have been divided into training and testing sets in a 6:4 ratio. The WOS_46985 dataset collects abstract information from articles published on the Web of Science with a total of 46,985 texts across seven original topics. The original WOS_46985 dataset was not divided into training and test sets, so we first randomly sampled the training and test set texts in a 6:4 ratio under the seven original topics. The AG_news dataset consists of four original topics, consisting of 127,600 news articles collected from more than 2000 news sources, and it has been divided into 120,000 training texts and 7600 test texts in a roughly 6:4 ratio.

### 4.2. LDA Model Training

We use the same data pre-processing approach for different datasets. Before training the LDA model on the documents, the text information of all documents is firstly processed for word splitting and stop word removal. We used the Lemminflect package [24] in Python for word formation reduction during the word separation. The stop word list was used to remove stop words from Google’s 2014 stop word list. In addition, we also removed words with a frequency of less than 20. The original dataset is divided into 60% training set and 40% test set. In order to verify the effectiveness of our proposed optimal topic number selection method, we use the original 60% training set for model training and topic number selection and then use the remaining 40% test set for validation. Before training, we first randomly sampled 80% of the original 60% sample as the training set and the remaining 20% of the original training set as the model test set. By this process, we sampled a total of 30 times.

In this study, we use the Gensim package [25] in Python for LDA model training. The training parameters must be set before training. The first is the number of topics to be trained, which is denoted by *k*. The number of topics trained for the 20new_groups dataset ranges from five to 30. The number of topics trained for the WOS_46986 dataset ranges from two to 20. The number of topics trained for the AG_news dataset ranges from two to 20. The hyperparameter *α* indicates the sparsity of topics in the document, that is, the set of topics in which the document is expected to contain most of the topics; the larger the α, the greater the number of topics that can be contained in the document. *β* indicates the sparsity of words in the topics, the smaller *β* indicates the non-uniform distribution of words within the topics. According to Hagen (2018), when the number of topics is not very large, different initializations do not have a significant impact on topic variation [26]. This study finally set both α and *β* in all LDA training models as dynamic variables related to the number of topics, which is $\frac{1}{k}$. The number of iterations was set to 400.

When calculating the stability of the model, the first step is to select the number of words that represent each topic. According to the formula for calculating the stability, it is known that the lower ranked words have less influence on the final calculation result, but the more words there are, the larger the calculation volume is, so it is not necessary to include all the topic words in the calculation. Existing studies have not reached a consensus on how many words should be selected to represent a topic [11]. Take the 20news_groups dataset as an example; the first *t* words with the highest probability of each topic were selected to represent that topic, and the values of *t* were taken as 20, 50, 80, and 100. Table 5 shows the correlation coefficients of the average stability of the model with different number of words.

As can be seen from Table 5, the stability of the model under different numbers of topic words is highly correlated, and the highest average Pearson is reached when the number of topic words is 50, indicating that the first 50 words of each topic are most capable of covering the meaning of the topic under the remaining number of words. Therefore, this study finally uses the first 50 words with the highest probability of each topic to represent that topic.

### 4.3. Comparison of Experimental Results

In this section, we compare the optimal topic number selection method proposed in this paper with the several commonly used topic number selection methods such as perplexity, JS divergence, coherence, and HDP (Hierarchical Dirichlet Process) by using three different datasets, which are 20news_groups, WOS_46986, and AG_news. Among these methods, the smaller the value of both the optimal topic score and the perplexity score we propose, the better the classification of the topic model. In contrast, the larger the JS divergence and coherence scores, the better the classification of the topic model. Teh et al. (2006), on the other hand, improved the original LDA model by proposing Markov chain Monte Carlo algorithms for posterior inference in hierarchical Dirichlet process mixtures and describe applications to problems in information retrieval and text modeling [27].

In addition, we use an LDA model based on the word-embedding approach to further validate the applicability of our proposed method. Regarding the processing of the LDA model for the word-embedding approach, we use the word-embedding data provided by Pennington et al. (2014), which are named GloVe [28]. For computational reasons, we use the word-embedding data, glove.6B.50d.txt, where each word is represented by 50 dimensions. GloVe is an unsupervised learning algorithm for obtaining vector representations for words. Training is performed on aggregated global word–word co-occurrence statistics from a corpus. The advantage is that by representing words as vectors with multiple dimensions, it enables similar words to have similar vector representations and can improve the accuracy of text analysis and recognition. Before word-embedding LDA model training, we first need to obtain a vector representation of each word in the dictionary. Take the 20news_groups dataset as an example, a 50-dimensional vector representation of the dictionary is found in glove.6B.50d.txt, and a total of 9503×50-dimensional dictionaries for 9503 words are obtained. Second, calculate the word similarity. The cosine similarity is calculated for each word in the dictionary with each word in glove.6B.50d.txt. For computational reasons and to avoid introducing too many dissimilar words, we extract the top 20 words with similarity as the synonymous related words of the original word and generate a 9503×20-dimensional near-sense word dictionary. Third, the word expansion of the original 20news_groups documents based on the near-sense word dictionary will replace each word of each document with 20 words from the similar word dictionary. Due to the presence of a large number of short texts in 20news_groups, most of the words appear less frequently, resulting in the generated document feature matrix being too sparse. This leads to the difficulty for the original LDA model to cluster some documents with the same subject content but different word usage and low word frequency into the same class. However, the expansion of the original vocabulary with similar words by the word-embedding method can greatly reduce the sparsity of the original documents and improve the accuracy of LDA model classification. Since the data size of WOS_46986 and AG_news is large and the concentration of the text is relatively high, we processed the word embedding of these two datasets by taking the top ten words with the highest similarity.

#### 4.3.1. The Experimental Results of 20News_Groups

##### Comparison of Different Topics Number Selection Methods

**Figure 3:**
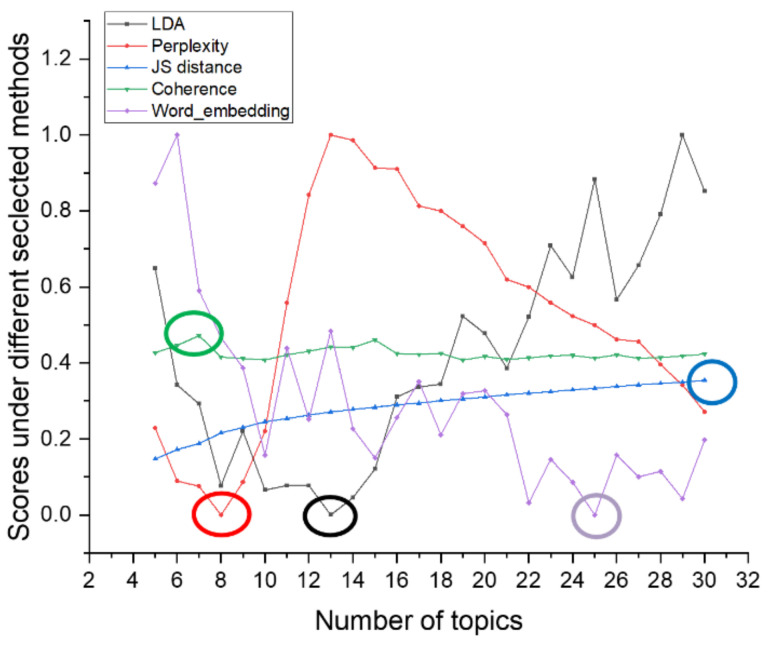
Scores under different selection methods.

The JS divergence and coherence scores are between 0 and 1. Compared with them, the topic superiority score and perplexity scores have larger values. To better show the differences in topic number selection of different methods in the figure, we normalize the topic superiority score and perplexity scores. The results of different topic selection methods are shown in Figure 3.

As can be seen in Figure 3, the optimal number of topics for the 20news_groups training set derived from our proposed optimal topic number selection method is 13. In the range of five to 13 topics, the optimal topic score tends to decrease, while in the range of 13 to 30 topics, the optimal topic score tends to increase. It can be seen that the optimal number of topics selection method we propose exhibits an approximate U-shape in the range of topics from five to 30 topics. Since our approach is that the smaller the score of the model, the better the classification of the topics, based on this U-shaped result, we believe that it is possible to find the optimal value by applying our optimal topic number selection method within a certain range of topic numbers.

The optimal number of topics selected by the perplexity method is 8. The model perplexity score tends to increase in the range of topics selected from eight to 15, and it again shows a significant downward trend between topics selected from 15 to 30. Although the optimal number of topics selected by the perplexity method is eight in the range of five to 30, the trend of a sharp decrease in the perplexity score as the number of topics rises cannot be ignored. Later, we will apply a real example of patent policy to test the perplexity method and find that the perplexity score decreases as the number of topics increases, which also proves this point.

The JS divergence method shows an increasing and non-converging trend in the scores calculated in the interval from five to 30 topics, so the optimal number of topics chosen for this method in this interval is 30.

The calculated score of the coherence method is relatively smooth in the interval from five to 30 topics, and the optimal number of topics selected is seven, but it also has a low peak at 15 topics.

We used the Tomotopy package [29] in Python to perform the HDP selection method, with each parameter set to its default value, and ended up with an optimal number of 59 topics.

The optimal number of topics derived from our proposed method in the word-embedding LDA model is 25. In the range of topics from five to 25, the calculated scores show a significant decreasing trend.

From the above analysis, it can be seen that the score curve obtained by our proposed optimal topic number selection method is closest to the U-shape, and therefore, the optimal value is most likely to exist. The optimal topic number obtained on the use of the word-embedding LDA model is closest to the original value. In contrast, other topic number selection methods may have the following problems.

First, the perplexity method has the possibility that the calculated score decreases as the number of topics increases, leading to the final inability to select the optimal number of topics.

Second, the scores obtained by the JS divergence method increase with the number of topics, which makes it difficult to converge, and it also suffers from the problem that the final number of selected topics is too large.

Third, the score curve calculated by the coherence method is too smooth, and multiple optimal values may appear.

Fourth, when selecting the number of topics for the 20news_groups dataset, we found that the number of topics selected by the HDP method was too large. However, the HDP method is an improvement of the original LDA training model by mapping the text vector to the topic feature space to obtain a three-layer model of document–topic–feature items, and then, the optimal topics can be trained automatically. We find that there are still two problems in the application of the HDP method. The features that a reasonable topic classification should satisfy are not fully considered, such as the four features mentioned in our method. This leads to the fact that the optimal number of topics derived from the HDP method may be very different from the actual number of topics. In addition, the HDP method is difficult to be applied to other improved LDA models or some other text classification models constructed based on word and topic distributions because it is an improvement of the training process of the original LDA model.

According to the results of selecting the number of topics in the 20news_groups dataset, it can be seen that the number of topics selected by our proposed method is closest to the original 20 topics. If the number of topics selected is too large, it may lead to too much subdivision of topics and too much similarity between topics, while if the number of subjects selected is too small, it may lead to some topics that should be separated being merged.

##### Clustering Effect Comparison

To further verify the effectiveness of our proposed optimal topic number selection method, this section uses the T-SNE package [30] in Python to visualize and compare the test set and training set document clustering effects under the LDA topic models selected by different methods.

According to the LDA document generation formula, the generation probability of each word in each document is shown in Equation (11).

(11) $$p\left(w|d_{m}\right)=\sum_{z=1}^{K}p\left(w|z\right)p\left(z|d_{m}\right)=\sum_{z=1}^{K}\phi_{m}\theta_{m}$$

where $\phi_{m}=p\left(w|z\right)$ denotes the probability of word *w* in the topic *z*. $\theta_{m}=p\left(z|d_{m}\right)$ denotes the probability of topic *z* in the *m*th document *d_m_*. Therefore, the generation probability of a document containing *n* word vectors *W* is shown in Equation (12).

(12) $$p\left(W|d_{m}\right)=\prod_{i=1}^{n}\sum_{z=1}^{K}p\left(w|z\right)p\left(z|d_{m}\right)=\prod_{i=1}^{n}\sum_{z=1}^{K}\phi_{m}\theta_{m}$$

After training the LDA model on the original test set of 20news_groups data in the previous section, we have obtained the “topic-word” probability distribution for these datasets with different number of topics *k*, which is $\phi_{m}$ in Equation (12). For the test set, we can calculate the “document-topic” probability distribution $\theta_{m}$ for each document based on the known “topic-word” probability distribution $\phi_{m}$.

In this paper, we use the Gibbs Sampling approach, which is commonly used in LDA models, to estimate the “document-topic” probability, as shown in Equation (13).

(13) $$p(z_{i}=k|{\vec{z}}_{\neg i},\vec{w})\propto p(z_{i}=k,w_{i}=t|{\vec{z}}_{\neg i},{\vec{w}}_{\neg i})\;=E(\theta_{mk})\times E(\phi_{kt})\;={\hat{\theta }}_{mk}\times {\hat{\phi }}_{kt}\;=\frac{n_{k,\neg i}^{\left(k\right)}+\alpha_{k}}{\sum_{k=1}^{K}(n_{k,\neg i}^{\left(k\right)}+\alpha_{k})}\times \frac{n_{k,\neg i}^{\left(t\right)}+\beta_{t}}{\sum_{k=1}^{K}(n_{k,\neg i}^{\left(t\right)}+\beta_{t})}$$

where ${\hat{\theta }}_{mk}$ and ${\hat{\phi }}_{kt}$ correspond to the “document-topic” probability estimation and “topic-word” posterior probability estimation in the LDA model. ¬i means remove the word with subscript *i*. $n_{k,\neg i}^{\left(k\right)}$ denotes the count of words belonging to topic k in the *m*th document after removing the words with subscript *i*. For the test set, ${\hat{\phi }}_{kt}$ is stable and invariant, so only ${\hat{\theta }}_{mk}=\frac{n_{k,\neg i}^{\left(k\right)}+\alpha_{k}}{\sum_{k=1}^{K}\left(n_{k,\neg i}^{\left(k\right)}+\alpha_{k}\right)}$ is needed to be estimated, and then, we can obtain the posterior probability of the topic to which each word in document *m* belongs. Finally, the “document-topic” probability of the test document can be calculated based on the statistics of the topics corresponding to each word after multiple sampling.

The specific steps are as follows.

Step1: Select one of the documents m in the test set and assign a random topic number *z* to each word *w* in this document. Set the initial value of the hyperparameter *α*. In this paper, the initial value of *α* is set to *1/k*.

Step2: The probability of each word corresponding to topic *z* is estimated using the Gibbs Sampling method, and then, the topic number corresponding to each word is updated.

Step2.1: Estimation of the current topic distribution of document m, which is ${\hat{\theta }}_{mk}=\frac{n_{k,\neg i}^{\left(k\right)}+\alpha_{k}}{\sum_{k=1}^{K}\left(n_{k,\neg i}^{\left(k\right)}+\alpha_{k}\right)}$.

Step2.2: Calculate the probability that each word in document *m* corresponds to the currently assigned topic, that is, $p(z_{i}=k|{\vec{z}}_{\neg i},\vec{w})={\hat{\theta }}_{mk}\times {\hat{\phi }}_{kt}$, where ${\hat{\theta }}_{mk}$ is estimated from Step2.1, and the “document-topic” probability ${\hat{\phi }}_{kt}$ is derived from the training set.

Step2.3: The topics to which each word belongs are reassigned according to the current topic probability $p(z_{i}=k|{\vec{z}}_{\neg i},\vec{w})$ corresponding to each word.

Step3: Repeat Step2 until Gibbs Sampling convergence reaches a steady state, that is, the estimate of $p(z_{i}=k|{\vec{z}}_{\neg i},\vec{w})$ is stable, at which point we consider the assignment of topics to document m reasonable.

Step4: The “document-topic” probability distribution of document *m* is obtained by counting the frequency distribution of the topic to which each word in document *m* belongs.

Step5: Repeat Step1 to Step4 for each document in the test set to obtain the “document-topic” probability for each document.

Each document in the training and test sets is represented by a k-dimensional “document-topic” probability distribution, which is used to draw a topic clustering map of the documents. To better compare the clustering effect of the documents in the training and test sets, we first use a binary variable to label the documents as training or test documents, respectively. Then, the documents in the training and test sets are clustered together based on the k-dimensional “document-topic” probability distribution of each document. Finally, the clustering graphs of the documents in the training set and test set are displayed separately according to the document IDs. This enables the borders of the clustering graphs of the training and test sets to be consistent, allowing us to distinguish the performance of the topic models in the training and test sets more clearly. This processing allows the distribution of the clusters in the training and test sets to be consistent across the range, allowing us to analyze the performance of the topic models on both the training and test sets more clearly.

Among the original topics in the 20news_groups dataset, some of them have widely different content (e.g., comp.sys.mac.hardware and rec.sport.hockey belong to the computer and sports domains, respectively), while others have more similar content (e.g., rec.sport.hockey and rec.sport. baseball belong to the same field of sports). In this section, we compare the clustering effect of documents with similar content and the clustering effect of documents with more different content.

**Figure 4:**
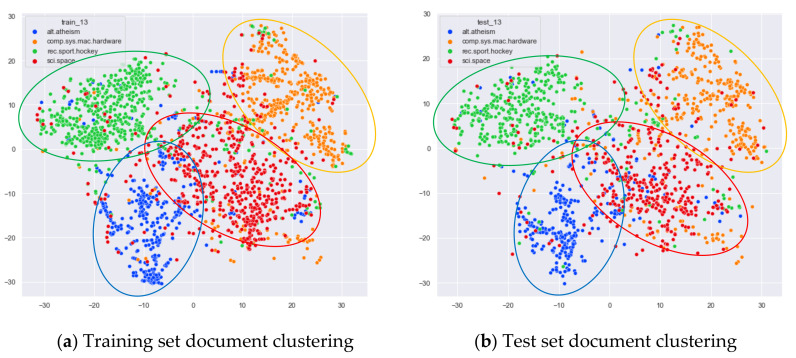
Clustering of training and test sets by LDA model with 13 topics (optimal topic number selection method).

**Figure 5:**
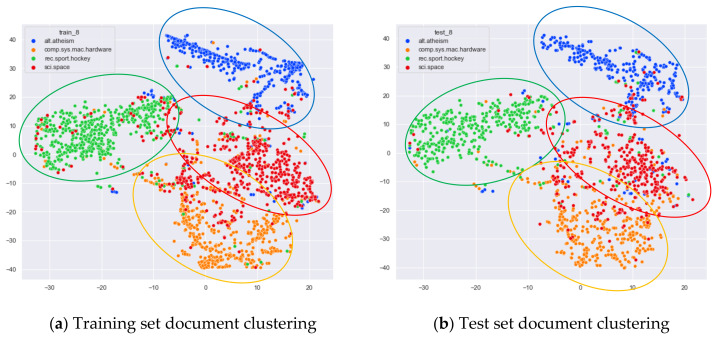
Clustering of training and test sets by LDA model with eight topics (perplexity method).

**Figure 6:**
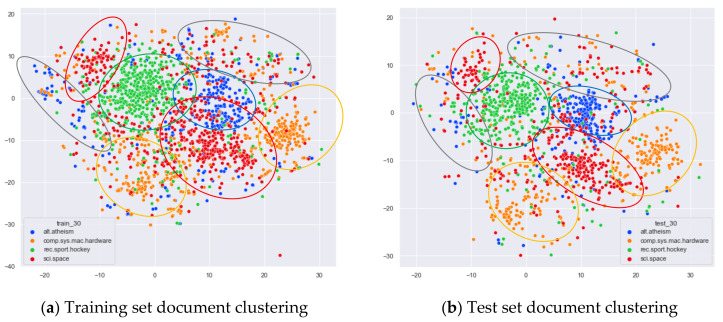
Clustering of training and test sets by the LDA model with 30 topics (JS divergence).

**Figure 7:**
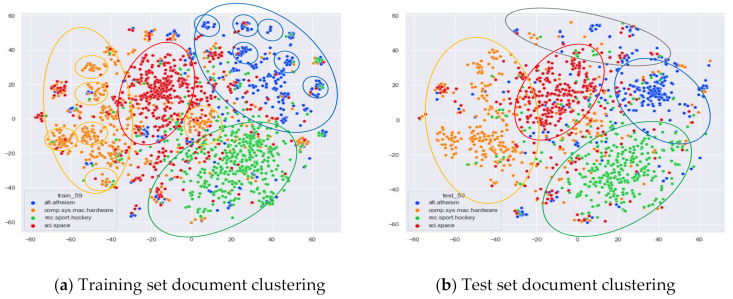
Clustering of training and test sets by LDA model with 59 topics (HDP method).

First, we select documents with large differences in topic content for clustering comparison, which are documents under the four original topics of alt.atheism, comp.sys.mac.hardware, rec.sport.hockey, and sci.space. We compare the clustering of the training and test set documents under 13 topics (LDA) and 25 topics (word-embedding LDA) selected by our proposed optimal topic selection method, 8 topics selected by the perplexity method (the number of topics selected by the coherence method is seven, which is basically the same as the number of topics selected by the perplexity method, so we only use the LDA training model with eight topics for analysis), 30 topics selected by the JS divergence method, and 59 topics selected by the HDP method, as shown in Figure 4, Figure 5, Figure 6 and Figure 7. Each dot in the figure represents a document, the color of the dot indicates the original topic it belongs to, and the elliptical box line indicates the clustering result of the LDA model under that number of topics.

From Figure 4a, we can see that the LDA model with 13 topics selected by the optimal topic number selection method is able to classify well the documents originally belonging to the four topics of alt.atheism, comp.sys.mac.hardware, rec.sport.hockey, and sci.space in the original training set. As can be seen in Figure 4b, for the test documents not involved in the LDA model training, the LDA model under 13 topics is also able to reasonably classify their topics, and the clustering results are basically consistent with the results of the training set in Figure 4a.

Figure 5a shows that the LDA model under eight topics selected by the perplexity method is able to classify well those documents that originally belong to the four topics of alt.atheism, comp.sys.mac.hardware, rec.sport.hockey, and sci.space in the original training set. As can be seen in Figure 5b, the LDA model with eight topics is also able to reasonably classify the topics for the test documents that did not participate in the LDA model training, and the clustering results are basically consistent with the results of the training set in Figure 5a.

From Figure 6a, it can be seen that the LDA model under 30 topics selected by the JS divergence method can cluster the documents in the training set in a relatively reasonable way, but it appears that those documents that originally belong to the same topic are divided into multiple classes. Documents that originally belonged to the category comp.sys.mac.hardware were clustered into two categories, as shown in the orange elliptic boxed line. Documents that originally belonged to the category sci.space were clustered into two categories, as shown in the red elliptic boxed line. The LDA model under 30 topics shows more fuzzy regions when clustering document topics, as shown in the gray elliptic region in Figure 6a. The clustering of the test set documents in Figure 6b is generally consistent with the clustering performance of the training set in Figure 6a.

From Figure 7a, we can see that the LDA model under 59 topics selected by the HDP method is able to perform some topic classification of the documents under the original training set. However, this classification suffers from the problem of dividing documents that originally belong to the same topic into multiple small clusters. This is due to the excessive number of topics selected by the HDP method, which results in documents that originally belong to one topic being divided into multiple topics. This excessive topic division can lead to a reduction in the ability of the LDA topic model to perform topic division for untrained documents. As shown in Figure 7b, when using the test documents that did not participate in the training for topic clustering, the clusters appear too scattered, and the boundaries between topics and themes are too blurred. In particular, the documents under the topic alt.theism still show some clustering effect in the training set, but the clustering effect in the test set is greatly reduced, and even some more fuzzy areas appear; see the gray elliptic area in Figure 7b.

**Figure 8:**
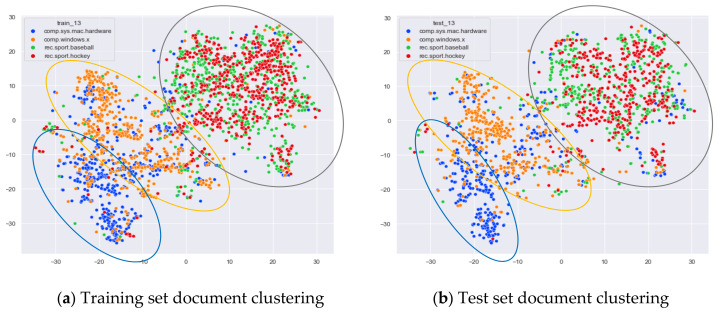
Clustering of training and test sets by LDA model with 13 topics (optimal topic number selection method).

**Figure 9:**
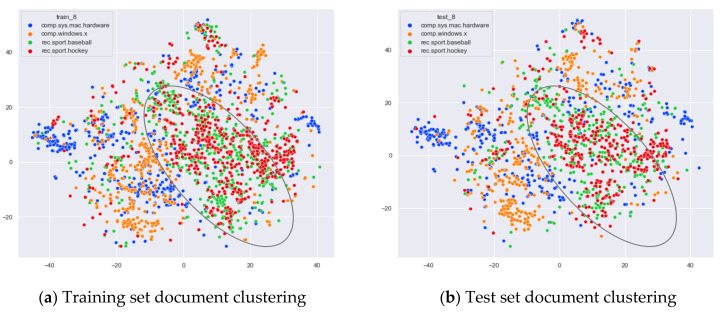
Clustering of training and test sets by LDA model with eight topics (perplexity method).

**Figure 10:**
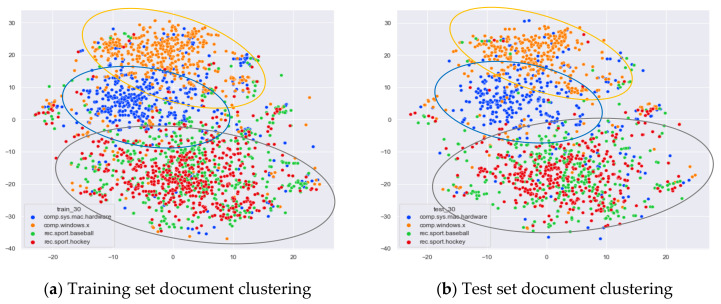
Clustering of training and test sets by LDA model with 30 topics (JS divergence).

**Figure 11:**
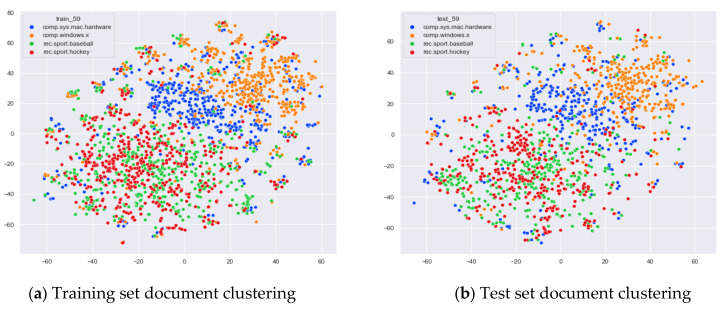
Clustering of training and test sets by LDA model with 59 topics (HDP method).

Then, we would like to further observe the clustering effect of the LDA model for documents with more similar topics under different topic number selection methods. We have selected the documents under the four original topics comp.sys.mac.hardware, comp.windows.x, rec.sport.baseball, and rec.sport.hockey. The documents under these four original classes were chosen because comp.sys.mac.hardware and comp.windows.x both belong to the computer domain, and rec.sport.baseball and rec.sport.hockey both belong to the sports domain, as shown in Figure 8, Figure 9, Figure 10 and Figure 11.

From Figure 8a, we can see that the LDA model with 13 topics selected by the optimal topic selection method clusters the documents belonging to the four topics of comp.sys.mac.hardware, comp.windows.x, rec.sport.baseball, and rec.sport.hockey under the original training set into three categories, where the categories comp.sys.mac.hardware and comp.windows.x can be better divided into two categories, while rec.sport.baseball and rec.sport.hockey are clustered into one category. The clustering results in Figure 8b for the test documents that were not involved in the LDA model training are consistent with the results of the training set in Figure 8a.

From Figure 9a, we can see that the LDA model with eight topics selected by the perplexity method does not achieve good clustering for the documents belonging to the four original topics of comp.sys.mac.hardware, comp.windows.x, rec.sport.baseball, and rec.sport.hockey in the original training set. The demarcation of documents under the comp.sys.mac.hardware and comp.windows.x themes is blurred, and documents under the rec.sport.baseball and rec.sport.hockey themes are clustered into one category. The clustering results in Figure 9b for the test documents that were not involved in the LDA model training are consistent with the results of the training set in Figure 9a.

From Figure 10a, we can see that the LDA model with 30 topics selected by the JS divergence method clusters the documents belonging to the four topics of comp.sys.mac.hardware, comp.windows.x, rec.sport.baseball, and rec.sport.hockey under the original training set into three categories, where the categories comp.sys.mac.hardware and comp.windows.x can be better divided into two categories, while rec.sport.baseball and rec.sport.hockey are clustered into one category. The clustering results in Figure 10b for the test documents that were not involved in the LDA model training are consistent with the results of the training set in Figure 10a.

As can be seen in Figure 11a, the LDA model for the 59 topics selected by the HDP method clusters those training set documents belonging to the four original topics of comp.sys.mac.hardware, comp.windows.x, rec.sport.baseball, and rec.sport.hockey into roughly three categories. The documents belonging to the categories comp.sys.mac.hardware and comp.windows.x can be better divided into two categories, while the documents belonging to rec.sport.baseball and rec.sport.hockey are clustered into one category. While this result is consistent with the LDA training model with 13 topics and 30 topics, the LDA model with 59 topics clearly clusters each topic into more subclasses. As can be seen from Figure 11b, the clustering effect for the test documents that did not participate in the training is basically the same as the training set, but the original training model divides too many topics, resulting in the clustering effect of the test set documents under the number of 59 topics not being as clear as the boundaries of the clusters with 13 topics and 30 topics.

Finally, the performance of our proposed optimal topic number selection method on the word-embedding LDA model will be verified. In addition to choosing documents from the same original categories as before for comparison, we added a categorical comparison of documents from four topics: comp.graphics, comp.sys.mac.hardware, rec.autos, and sci.space.

**Figure 12:**
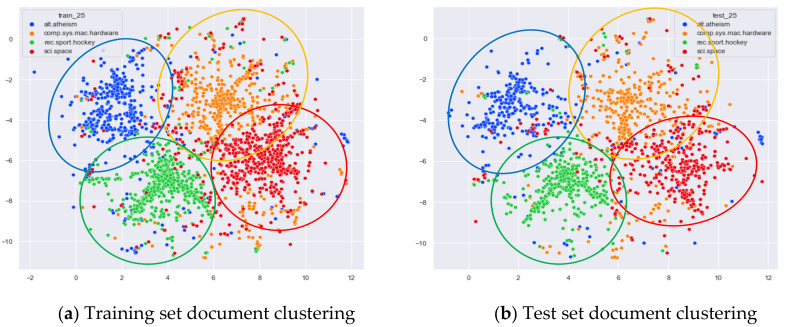
Clustering of training and test sets by word-embedding LDA model with 25 topics (optimal topic number selection method).

From Figure 12a, we can see that the word-embedding LDA model with 25 topics selected by the optimal topic number selection method is able to classify the documents originally belonging to the four topics of alt.atheism, comp.sys.mac.hardware, rec.sport.hockey, and sci.space in the original training set well. As can be seen in Figure 12b, the word-embedding LDA model under 25 topics is also able to reasonably classify the topics for the test documents that did not participate in the word-embedding LDA model training, and the clustering results are generally consistent with those of the training set in Figure 12a. This result is consistent with the results of the original LDA model with a topic number of 13 selected by our method.

**Figure 13:**
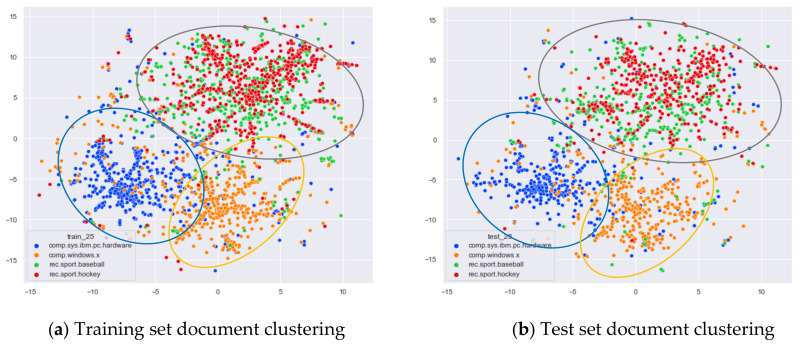
Clustering of training and test sets by word-embedding LDA model with 25 (optimal topic number selection method).

From Figure 13a, we can see that the word-embedding LDA model with 25 topics selected by the optimal topic selection method clustered the documents belonging to the four topics of comp.sys.mac.hardware, comp.windows.x, rec.sport.baseball, and rec.sport.hockey in the original training set into three categories. The categories comp.sys.mac.hardware and comp.windows.x were able to be better divided into two categories, while rec.sport.baseball and rec.sport.hockey were clustered into one category. As can be seen from Figure 13b, the clustering effect for the test documents that did not participate in the training is consistent with the training set. This result is consistent with the results of the original LDA model with a topic number of 13 selected by our method.

**Figure 14:**
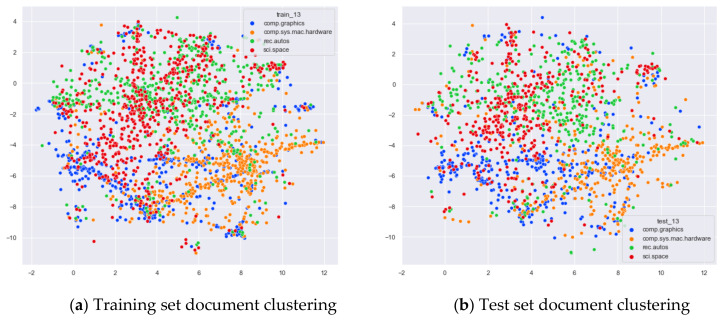
Clustering of training and test sets by word-embedding LDA model with13 (optimal topic number selection method).

As can be seen from Figure 14a, the document contents of the four original topics, comp.graphics, comp.sys.mac.hardware, rec.autos, and sci.space, are not identical, but the LDA models under the 13 topics selected by the optimal topic selection method are not clearly clustered for the original training set. The clustering effect on the test set is the same as that on the training set.

**Figure 15:**
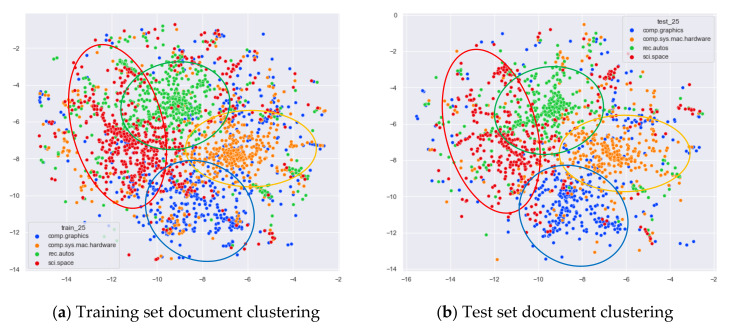
Clustering of training and test sets by word-embedding LDA model with 25 (optimal topic number selection method).

From Figure 15a, it can be seen that the word-embedding LDA model under 25 topics selected by the optimal number of topics selection method is able to classify documents originally belonging to the four topics of comp.graphics, comp.sys.mac.hardware, rec.autos, and sci.space under the original training set with better results than the original LDA model under LDA models with 13 topics, as shown in Figure 14a. As can be seen in Figure 15b, for the test documents that did not participate in the word-embedding LDA model training, the word-embedding LDA model under 25 topics is also able to classify their topics reasonably well, and the clustering results are basically consistent with those of the training set in Figure 15a.

#### 4.3.2. The Experimental Results of WOS_46986

##### Comparison of Different Topics Number Selection Methods

**Figure 16:**
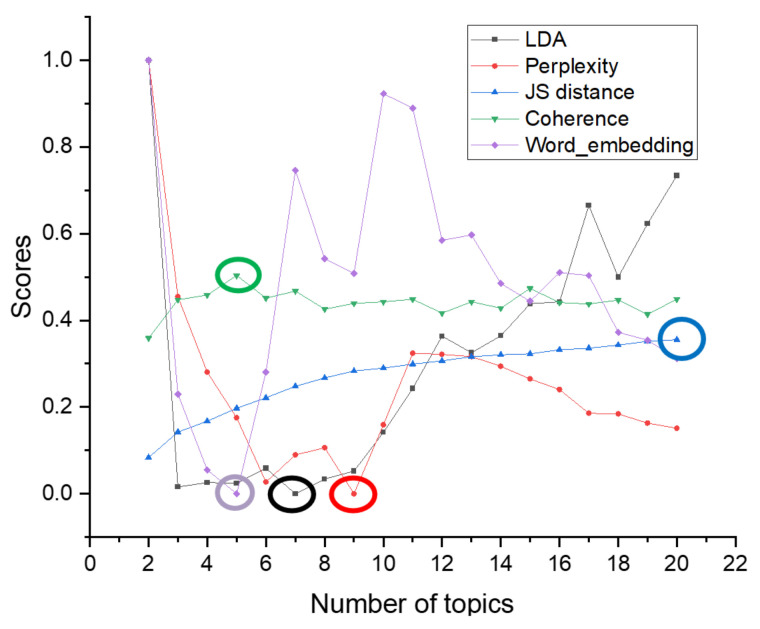
Scores under different selection methods.

In this section, we use the WOS_46986 dataset to compare the optimal topic number selection method proposed in this paper with several commonly used methods: the perplexity selection method, JS divergence selection method, and coherence selection method. We also include the results of the optimal topic number selection method applied to the word-embedding LDA model for the comparison. The topic model scores of different topic selection methods are shown in Figure 16.

As can be seen in Figure 16, the optimal topic number selection method proposed in this paper selects the number of topics in the LDA model as seven and selects the number of topics in the word-embedding LDA model as five. The optimal number of topics selected by the perplexity method is nine, the JS divergence method is 20, and the coherence selection method is five. The trends of the score curves calculated by the optimal topic number selection method, the perplexity selection method, the JS divergence method, and the coherence selection method in the WOS_46986 and 20new_groups datasets are generally consistent.

However, in the application of the word-embedding LDA model, the trend of the score curves calculated by the optimal topic number selection method in the WOS_46986 dataset is slightly different from that in the 20news_groups dataset. In the 20news_groups dataset, the score curve shows a clear downward trend in the range of five to 25 topics. In the WOS_46986 dataset, the score curve shows a decreasing trend in the range of two to five topics, an increasing trend in the range of five to 10 topics, and a decreasing trend again after the number of topics is greater than 10. There are two possible reasons for this discrepancy. First, the documents in the WOS_46986 dataset have relatively concentrated word expressions. Second, the original WOS_46986 dataset has further divided each topic into multiple subdomains under the seven main categories. The word-embedding method allows the words of each document to form more associative relationships on the subdomains, thus enabling further segmentation of the subdomains when the number of topics is further increased.

##### Clustering Effect Comparison

Among the original topics of the WOS_46986 dataset, there are some topics with large differences in document content, such as documents under the original topics of ECE, Psychology, and Biochemistry. There are also some topics with some overlap in document content, such as documents under the original topics Medical and Biochemistry, and documents under the original topics ECE (Electrical and Computer Engineering) and CS (Computer Science).

**Figure 17:**
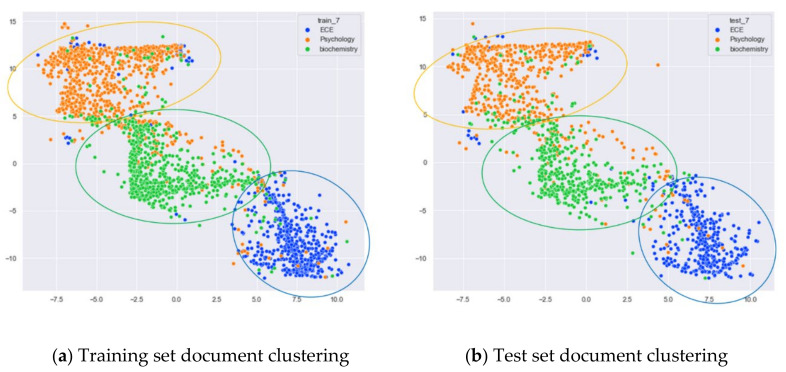
Clustering of training and test sets by LDA model with seven (optimal topic number selection method).

**Figure 18:**
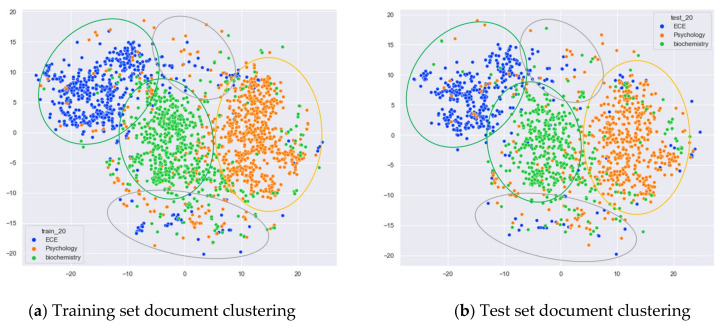
Clustering of training and test sets by LDA model with 20 (JS divergence).

**Figure 19:**
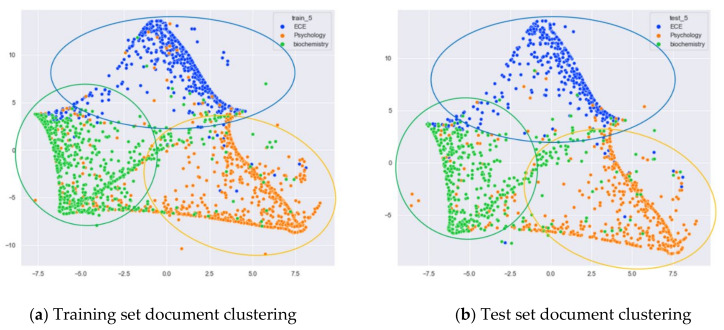
Clustering of training and test sets by word-embedding LDA model with 25 (optimal topic number selection method).

**Figure 20:**
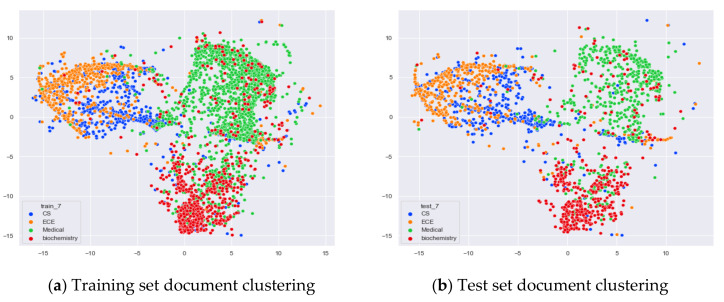
Clustering of training and test sets by LDA model with seven (optimal topic number selection method).

**Figure 21:**
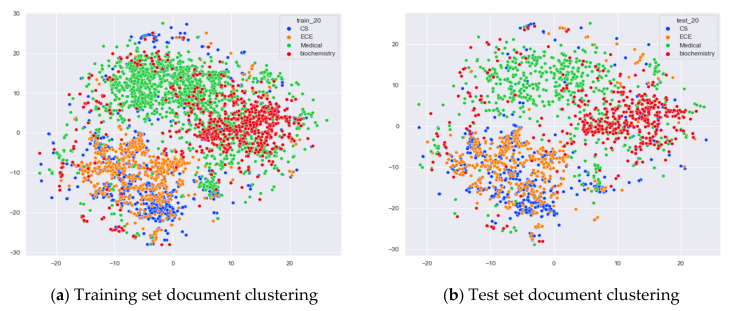
Clustering of training and test sets by LDA model with 20 (selected by JS divergence).

**Figure 22:**
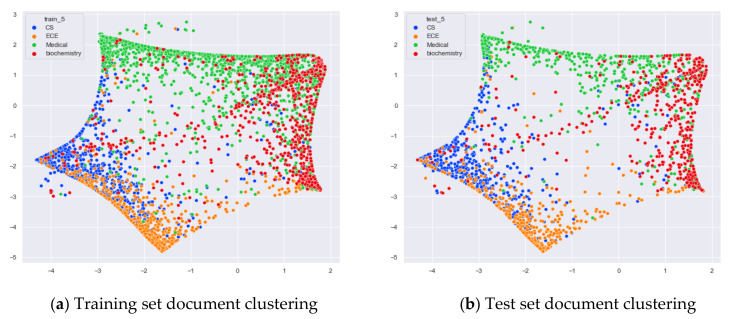
Clustering of training and test sets by word-embedding LDA model with five (optimal topic number selection method).

First, we selected three topics with large differences in content for clustering comparison, which are documents under the original topics of ECE (Electrical and Computer Engineering), Psychology, and Biochemistry. We compare the clustering of the training and test set documents under seven topics (LDA) and five topics (word-embedding LDA) selected by our proposed optimal topic selection method, and 20 topics selected by the JS divergence method. The number of topics selected by the perplexity method is nine, which is basically the same as the number of topics selected by the optimal topic selection method, so we only use the LDA training model with seven topics for analysis, as shown in Figure 17, Figure 18, Figure 19, Figure 20, Figure 21 and Figure 22. Each dot in the figure represents a document, the color of the dot indicates the original topic it belongs to, and the elliptical box line indicates the clustering result of the LDA model under that number of topics.

From Figure 17a, we can see that the LDA model with seven topics selected by the optimal topic number selection method is able to classify well the documents originally belonging to the three topics of ECE, Psychology, and Biochemistry in the original training set. As can be seen in Figure 17b, for the test documents not involved in the LDA model training, the LDA model under seven topics is also able to reasonably classify their topics, and the clustering results are basically consistent with the results of the training set in Figure 17a.

From Figure 18a, it can be seen that the LDA model with 20 topics selected by the JS divergence method is able to properly classify the documents originally belonging to the three topics of ECE, psychology, and biochemistry in the original training set. However, compared with the LDA model with seven topics, the clustering boundary under the LDA model with 20 topics is more blurred. As can be seen in Figure 18b, the clustering results of the LDA model under 20 topics for the test documents that did not participate in the training of the LDA model are generally consistent with the results of the training set in Figure 18a.

From Figure 19a, it can be seen that the word-embedding LDA model under five topics selected by the optimal topic number selection method is able to classify those documents originally belonging to the three topics of ECE, Psychology, and Biochemistry under the original training set well. As can be seen in Figure 19b, the word-embedding LDA model under five topics can also reasonably classify the topics for the test documents that did not participate in the training, and the clustering results are basically consistent with the results of the training set in Figure 19a.

Next, we further observe the classification effect of the LDA model for documents with more similar topic content under different numbers of topics. We selected documents under the original categories of CS, ECE, Medical, and Biochemistry. Among them, there are many documents with overlapping contents in CS and ECE topics, and some documents with overlapping contents in Medical and Biochemistry topics.

From Figure 20a, we can see that the LDA model with seven topics selected by the optimal topic number selection method divides the documents originally belonging to the four categories of CS (Computer Science), ECE (Electrical and Computer Engineering), Medical, and Biochemistry into three clusters. The documents under Medical and Biochemistry have some overlap, but the respective clusters can be roughly seen, while the documents originally belonging to the CS and ECE categories have more overlap. As can be seen in Figure 20b, the clustering results for the test documents that were not involved in the LDA model training are basically the same as the results of the training set in Figure 20a.

From Figure 21a, it can be seen that compared to the LDA model under seven topics, the LDA model with 20 topics has fuzzier clustering boundaries for the documents belonging to the four original categories of CS, ECE, Medical, and Biochemistry in the training set. As can be seen in Figure 4b, the clustering results of the LDA model with 20 topics for the test documents that did not participate in the training are generally consistent with the results of the training set in Figure 21a.

From Figure 22a, we can see that the LDA model with five topics selected by the optimal topic number selection method clusters the documents originally belonging to the four categories CS, ECE, Medical, and Biochemistry into four categories roughly. Although there is some overlap between the documents originally belonging to Medical and Biochemistry and the documents originally belonging to CS and ECE, the clustering effect of these categories is better than that of the LDA model with seven topics. As can be seen in Figure 22b, the clustering results for the test documents that were not involved in the training of the word-embedding LDA model are generally consistent with the results of the training set in Figure 22a.

#### 4.3.3. The Experimental Results of AG_News

##### Comparison of Different Topics Number Selection Methods

**Figure 23:**
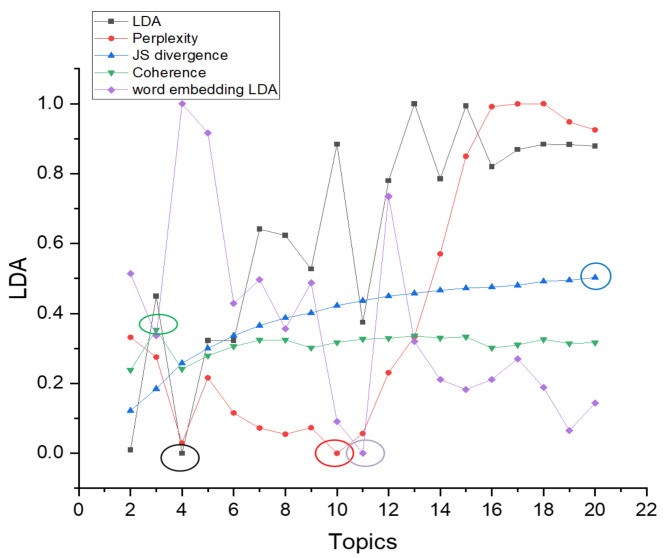
Scores under different selection methods.

In this section, we use the AG_news dataset to compare the optimal topic number selection method proposed in this paper with several commonly used methods: the perplexity selection method, JS divergence selection method, and coherence selection method. We also include the results of the optimal topic number selection method applied to the word-embedding LDA model for the comparison. The topic model scores of different topic selection methods are shown in Figure 23.

As can be seen in Figure 23, the optimal topic number selection method proposed in this paper selects the number of topics in the LDA model as four and selects the number of topics in the word-embedding LDA model as 11. The optimal number of topics selected by the perplexity method is 10, the JS divergence method is 20, and the coherence selection method is three.

##### Clustering Effect Comparison

The AG_news dataset includes four original themes: world, sports, business, and sci/tech. These four topics cover a wide range of content. Even for documents under the same topic, the content varies greatly. In this section, we compare the clustering effects of the LDA model with four topics and the word-embedding LDA model with 11 topics selected by the optimal topic number selection method, as well as the clustering effects of the LDA model with 10 topics selected by the perplexity method. The number of topics selected by the coherence method is three, which is basically the same as the number of topics selected by the optimal topic selection method, so we only use the LDA training model with four topics for analysis. The JS divergency method still suffers from the problem that the number of selected topics is too large, so its clustering effect is similar to that of the 20new_groups and WOS_46986 datasets, and we do not repeat the demonstration in this section.

**Figure 24:**
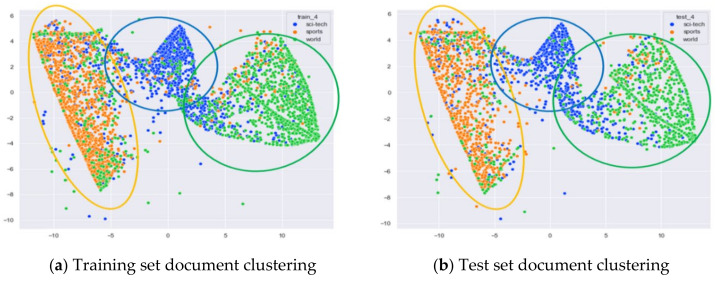
Clustering of training and test sets by LDA model with four topics (optimal topic number selection method).

**Figure 25:**
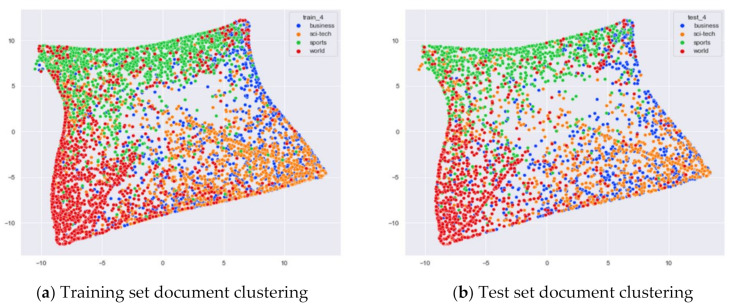
Clustering of training and test sets by LDA model with four topics (optimal topic number selection method).

Combining Figure 24 and Figure 25, it can be seen that the LDA model with four topics selected by the optimal topic number selection method can classify the documents under the three classes of Sci/Tech, Sports, and World in AG_news well. However, the clustering of documents under the original category of Business is not effective.

**Figure 26:**
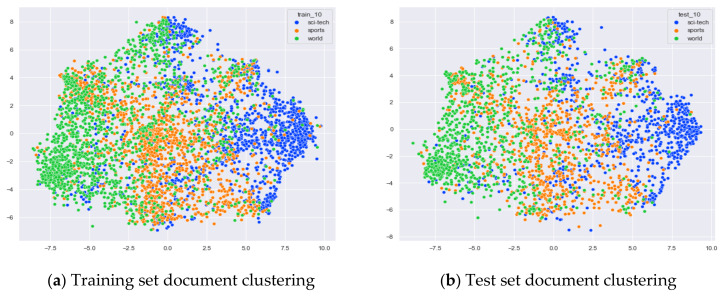
Clustering of training and test sets by LDA model with four topics (perplexity method).

**Figure 27:**
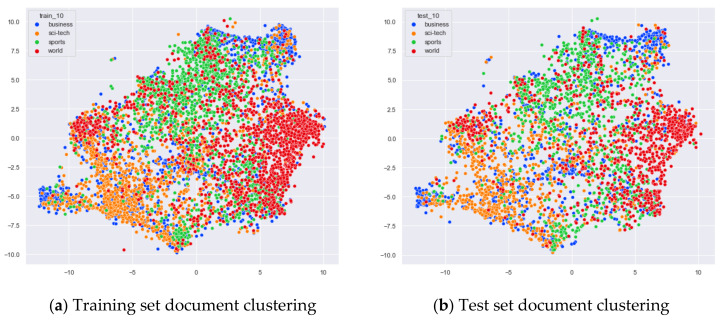
Clustering of training and test sets by LDA model with four topics (perplexity method).

Combining Figure 26 and Figure 27, it can be seen that the LDA model with 10 topics selected by the perplexity method can roughly classify the documents belonging to AG_news under the three classes of Sci/Tech, Sports and World, but the effect is not as good as that of the LDA model with four topics. At the same time, the clustering of documents belonging to the original category of business does not perform well.

**Figure 28:**
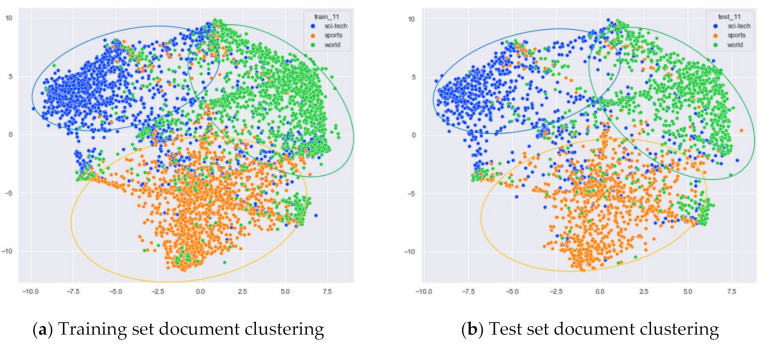
Clustering of training and test sets by word-embedding LDA model with 11 topics (optimal topic number selection method).

**Figure 29:**
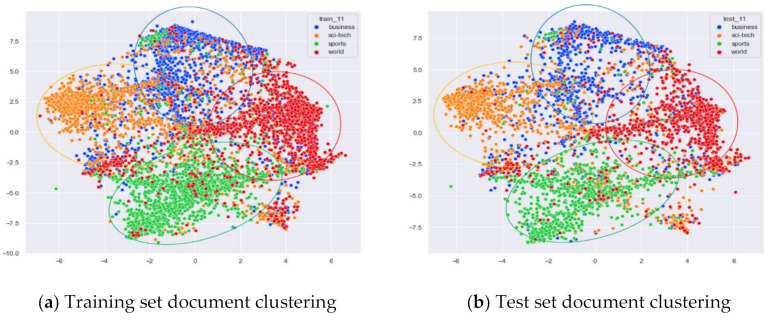
Clustering of training and test sets by LDA model with four topics (selected by optimal topic number selection method).

Combining Figure 28 and Figure 29, it can be seen that the word-embedding LDA model with 10 topics selected by the optimal number of topics selection method performs better than the two LDA models mentioned above. The word-embedding LDA model is not only able to classify the documents under the three categories of Sci/Tech, Sports, and World in AG_news but also to perform some clustering of the documents under business topics.

### 4.4. Comparison of Time Consumption

The experimental environment is a computer with Windows 10 Home Edition operating system, Intel (R) Core (TM) i7-11700 CPU, 2.50GHz, and 16GB RAM. We use different data sizes to compare the time consumption of different methods. The different data sizes are shown in Table 6.

As shown in Table 6, under the same dataset, the same amount of text is used for training the LDA model or the word-embedding LDA model. However, since the texts used for the word-embedding LDA model are processed for word expansion, the dictionary length, total word count, and average text length of these texts are larger than those used for LDA model training. In terms of dictionary length and the number of texts trained under the LDA model, the AG_news dataset has the longest dictionary and the largest number of texts, the WOS_46986 dataset is the next longest, and the 20news_groups dataset is the smallest. In terms of the size of the bag-of-words trained by the LDA model, the WOS_46986 dataset has the largest number of words, the AG_news dataset is the next largest, and the 20news_groups dataset is the smallest. In terms of the average text length under the LDA model, the WOS_46986 dataset has the longest average text length, the 20news_groups dataset has the second longest, and the AG_news dataset has the shortest. In the word-embedding LDA model, since the words in the 20news_groups dataset undergo a twenty times expansion process, while the WOS_46986 dataset and AG_news dataset are both ten times expanded, the dictionary length, word pocket size, and average text length of the expanded 20new_groups dataset are the largest. It can be seen that although the AG_news dataset has the largest number of texts, the dictionary of this dataset is long and the text content is short, resulting in most of the text content within the dataset being sparse. In contrast, the text content of the WOS_46986 dataset is more focused. The different text sizes of the datasets will result in different time consumption for model training and topic number selection, as shown in Figure 16.

**Figure 30:**
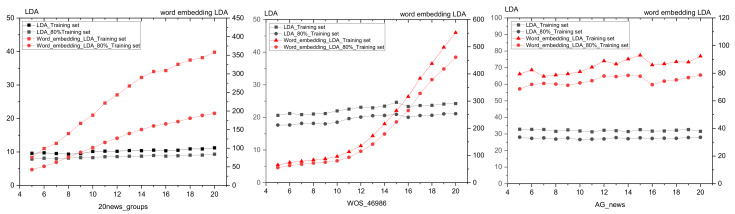
LDA and word-embedding LDA model training time consumption of three different datasets (the time consumption of the LDA model corresponds to the left *Y*-axis and the word-embedding LDA time consumption corresponds to the right *Y*-axis. Time unit: seconds).

The training speed of the LDA model is related to several factors. First, the larger the number of topics trained, the more time it takes. As shown in Figure 30, the time consumption of each data set increases with the *X*-axis coordinate. Second, the longer the average text length, the more time it takes. For example, although the word-embedding LDA model for the AG_news dataset has a larger bag-of-words size than the word-embedding dataset of 20news_groups and has the largest number of documents, its training time consumption is relatively small. Third, the larger the bag-of-words size, the more time consuming in the training, as can be seen from the comparison results of the red and black lines in Figure 30. Fourth, when the bag-of-words sizes are similar, the number of texts has a higher impact on the training time than the text length. For example, the 80% WOS_46986 dataset contains 19,199,000 words in the bag-of-words of the word-embedded text, and the 100% 20news_groups dataset contains 19,494,540 words in the bag-of-words of the word-embedded text, which are similar to each other. The average text length of the WOS_46986 dataset is 849.55 words smaller than the 1723.05 words of the 20news_groups dataset, but the WOS_46986 dataset contains 28,190 texts higher than the 11,314 texts of the 20news_groups dataset. Ultimately, the WOS_46986 dataset takes more time to train than the 20news_groups dataset, and this phenomenon becomes more apparent as the number of training topics increases.

**Figure 31:**
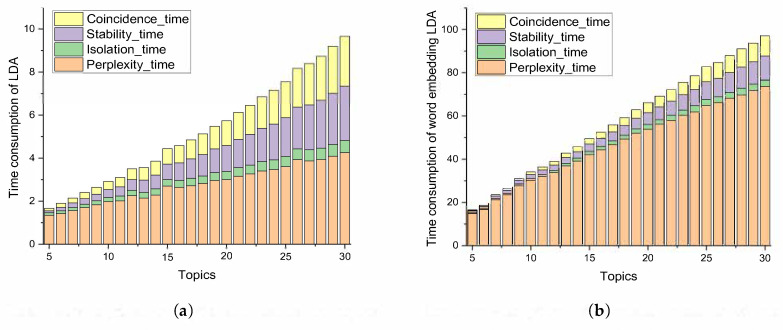
Comparison of training time of optimal number of topics selection method in LDA and word-embedding LDA (the areas in different colors in the figure indicate the computational time consumption for each index of our proposed method, and the total training time consumption is the sum of the areas of all bars. Time unit: seconds). (**a**) Time consumption of optimal number of topics selection method on LDA model. (**b**) Time consumption of optimal number of topics selection method on word-embedding LDA model.

The next discussion is the computational time consumption of different topic number selection methods for different data sizes. First, the computation time of the four dimensions of our proposed optimal topic number selection method is shown in Figure 31.

**Figure 32:**
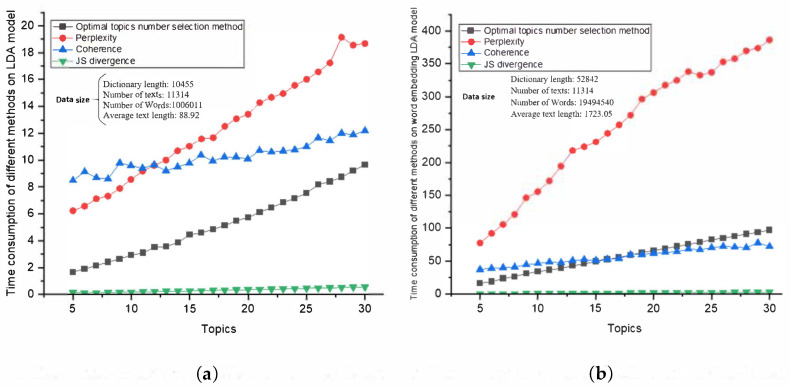
Comparison of time consumption of different topic number selection methods (time unit: seconds). (**a**) Time consumption of different methods on LDA model. (**b**) Time consumption of different methods on word-embedding LDA model.

It can be seen from Figure 31 that the computation time of the optimal topic number selection method on the word-embedding LDA model is higher than that on the ordinary LDA model. The time difference is the largest for the perplexity index, which is due to the fact that the perplexity calculation is related to the length of the text. It can also be seen from Figure 31 that the computation time of the optimal topic number selection method increases significantly as the number of topics increases. Figure 32 shows the computational time consumption of different topic selection methods for different dataset sizes.

**Figure 33:**
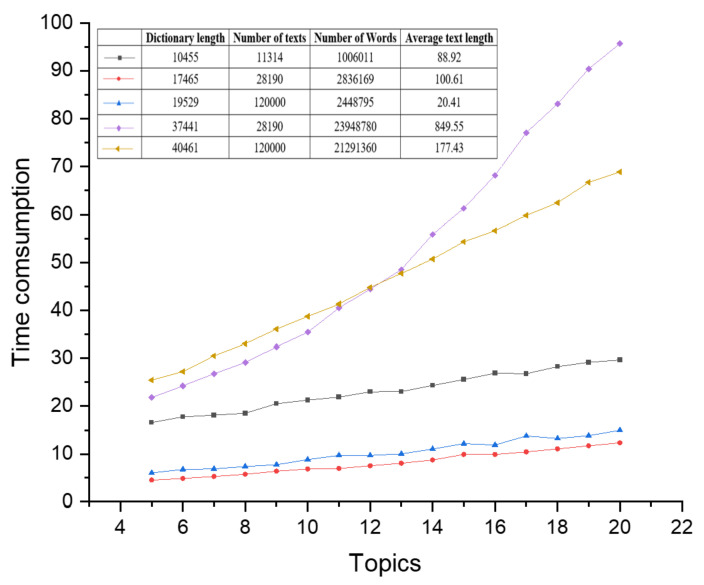
The computational time consumption of the optimal topic number selection method on different size datasets (time unit: seconds).

The time consumptions shown in Figure 32 are all for one sample training. From Figure 32, it can be seen that the computational time consumed by different topic number selection methods on both LDA models increases as the number of topics increases. Overall, the JS divergence method has the shortest computational time consumption on both LDA models. As seen in Figure 32a, the optimal topic number selection method takes less time than the perplexity and coherence methods for the topic number selection of the original LDA model. As seen in Figure 32b, the optimal topic number selection method takes less computational time than the perplexity method for the selection of topic numbers for the word-embedding LDA model. At the same time, when the number of trained topics is less than 15, the optimal topic selection method takes less time than the coherence method. When the number of trained topics is greater than 15, the optimal topic selection method takes more time than the coherence method. Figure 33 shows the computational time consumption of the optimal topic number selection method on different size datasets.

As can be seen from Figure 33, the overall computation time of the optimal topic selection method increases as the number of trained topics and the size of the data increases. In terms of data size differences, when the dictionary length and bag-of-words size are close (as shown by the lines in red and blue), the number of texts has a greater impact on computational time consumption than the text length. Relatively speaking, the bag-of-words size has the greatest impact on computational time consumption. For example, except for the smaller size of the bag-of-words, the data shown in the red line are larger in dictionary length, number of texts, and average text length than those in the black line. The result is that the data in the black line take more time to compute than the data in the red line. In addition, the effect of average text length on computational time consumption is greater when the number of trained topics is higher. For example, the purple and yellow lines are about the same size in terms of dictionary length and bag-of-words size. When the number of trained topics is less than 12, the data in the yellow line take more time to train than the purple line due to the higher number of texts. However, when the number of trained topics is greater than 12, the training time for the purple line exceeds that of the yellow line due to the longer average text length.

From the above, it is clear that although our proposed method is constructed to consider reasonable topic selection from four indicators, while several other methods consider only one aspect of topic classification, the time consumed of our proposed method is in the middle level in terms of the time consumed for one-time computation. In addition, it should be noted that if we want to obtain a more reliable optimal number of topic selection, we also need to perform multiple sampling, which will increase the computational time consumption of the optimal topic selection method.

In summary, our proposed optimal topic number selection method has several advantages. First, in terms of the number of topics selected, the optimal topic number selection method can better select the appropriate number of topics for the LDA model. Compared with several other topic number selection methods, the optimal number of topics derived from our method is closest to the real number of topics. In our example, the number of topics selected by the perplexity method and the coherence method is much smaller than the real number of topics. The optimal number of topics selected by the JS divergence method is at the maximum boundary of the topic selection interval, which makes us skeptical about whether this selected number of topics is optimal. The optimal number of topics selected by the HDP method is much larger than the true number of topics.

Second, from the score curves, the score curve of the number of topics derived from our proposed optimal topic number selection method is closest to a U-shaped curve, which makes it possible to find the optimal extreme value point in the score curve. On the one hand, the score curve derived from the perplexity method has an optimal point at the position where the number of topics is small. However, when the number of topics exceeds a certain threshold, the perplexity value decreases sharply with the increase in the number of topics, so the perplexity method may have the problem that the larger the number of topics selected, the lower the perplexity value, and the curve is difficult to converge. The topic number score curve derived from the coherence method presents a smooth fluctuating state, and the state of this curve is less reliable than the U-shaped curve obtained by the optimal topic number selection method in finding the optimal value. The curves derived from the JS divergence method, on the other hand, exhibit the problem of failure to converge as the number of topics increases.

Third, from the clustering graphs under different topic numbers, the optimal topic number selection method and the perplexity method are able to classify documents with large differences in content clearly. The optimal number of topics selected by the JS divergence method is large, and there is the problem of overclassifying content that is originally the same topic. The HDP method selects the largest number of topics, which leads it to divide the original same topic content into multiple subclasses, similar to the state of model overfitting in the training set, which leads the model to fail to perform good classification in the test set instead. For documents with small differences in content, the LDA topic model selected by the optimal number of topics selection method and the JS divergence method can classify those documents that are similar in content but still have some differences better, but it is difficult to achieve good classification for documents with too similar content. In the example of this paper, the number of topics selected by the perplexity method is too small, making it impossible to classify documents with similar content. Although the number of topics selected by the HDP method is larger, it is still unable to effectively divide documents with similar topics and suffers from the problem of topic over division. In addition, the HDP method is difficult to apply to other topic classification models since it is an improvement of the LDA model.

Fourth, although the number of topics selected by our proposed optimal topic number selection method cannot be exactly equal to the real number of topics, and there is the problem of difficulty in classifying topics for documents with similar content, this problem is in fact due to the original defect of the LDA model. The purpose of our proposed method is to evaluate LDA training models with different numbers of topics and to select the optimal one from them. It has been demonstrated by the above examples that this method can do this. However, our optimal topic number selection method does not change the defects of the original LDA topic model. If the original LDA model is unable to classify similar documents in any case, then our optimal topic number selection method is unable to do so, either. We use an improved word-embedding LDA model to further validate the applicability of our proposed method. It turns out that our proposed method is also applicable to this improved LDA model. Since the word-embedding method can compensate for the problem that the original document word expressions are too sparse to be clustered, it makes the final selected word-embedding LDA model have better clustering effect than the original LDA model.

In the subsequent sections, we will also use Chinese patent policy texts as real cases for LDA topic classification, and use our proposed optimal topic number selection method for topic division and calculation as further validation.

## 5. Experiment and Analysis for Real Data

### 5.1. Data and Pre-Processing

**Figure 34:**
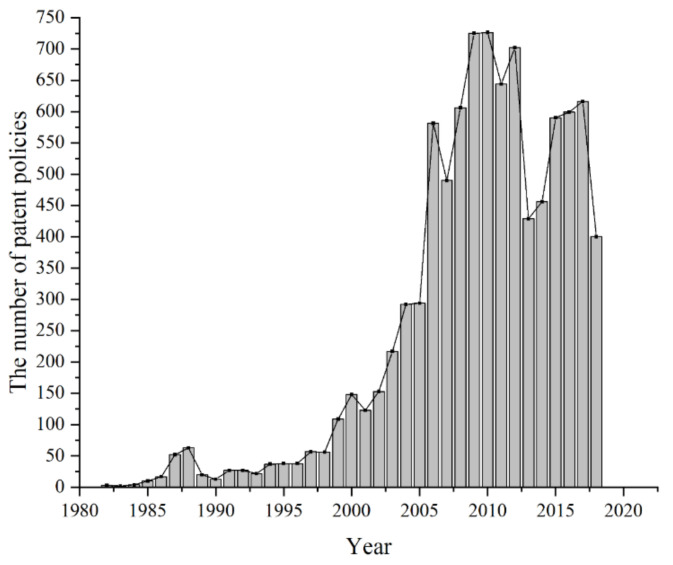
Number of patent policies in China from 1980 to 2018.

The policy texts involved in this study are obtained from the Wolters Kluwer Information Database and Peking University Law Database. The scope of collection is patent policy texts from all provinces and cities in China (excluding Hong Kong, Macau and Taiwan). Since some patent policies are included in other policy texts, in order to collect patent policies as extensively as possible, this study retrieved all policy texts with one of the following keywords in the title: patent, intellectual property, science and technology, innovation, and all policy texts with the word patent in the full text in the two databases. After removing duplicate texts and texts with less than 20 words of content, there were 9286 valid patent policy texts remaining, ranging from 1982 to 2018. As shown in Figure 34, the number of total patent policies in China is low between 1980 and 1999 (start-up period), shows a faster growth from 2000 to 2008 (growth period), and fluctuates somewhat from 2009 to 2018 (fluctuation period).

Before conducting text analysis, the 9286 policy texts are first pre-processed. The steps are as follows:

Step 1: Split words. Use Python’s Jieba package [31] to split all the text into words.

Step 2: Merge synonyms. For example, the names of universities are combined into universities; the names of banks are combined into banks; award rankings and grades are combined into award grades; technology parks with regional names are unified, etc.

Step 3: Remove stop words. Punctuation, graphics, non-Chinese characters, auxiliary words, and words with a word frequency less than or equal to 10 are removed. Due to the wide range of policies collected in this study, some policies also contain topics that are not related to patents or technologies. In order to avoid producing unnecessary topics during LDA model training, this study also removes this part of irrelevant words. The final number of valid words obtained is 13,309.

### 5.2. LDA Model Training

The specific training process is consistent with that described in Section 4. First, the cleaned 9286 patent policy texts are trained to generate LDA models under different topic numbers *k*. Then, the ratio of the training set and test set is set as 8:2, where 80% of the documents from all policy texts are randomly selected as the training set and the remaining 20% are selected as the test set for 30 times. In this part, the first *t* words with the highest probability of each topic were selected to represent that topic, and the values of *t* were taken as 20, 50, 80, and 100. Table 7 shows the correlation coefficients of the average stability of the model with different numbers of words.

As can be seen from Table 7, the stability of the model under different numbers of topic words is highly correlated, and the highest average Pearson is reached when the number of topic words is 50, indicating that first 50 words of each topic are most capable of covering the meaning of the topic under the remaining number of words. Therefore, this study finally uses the first 50 words with the highest probability of each topic to represent that topic.

Finally, the perplexity, isolation, stability, and coincidence of the model are calculated according to the steps in Section 3.

### 5.3. Experimental Results and Comparative Analysis

#### 5.3.1. Comparison of Optimal Number of Topics

The values calculated by the different topic selection methods are normalized as shown in Figure 20. The optimal topic number selection method proposed in this paper selects the number of topics in the LDA model as 54, while the perplexity and JS divergence method are both 70.

**Figure 35:**
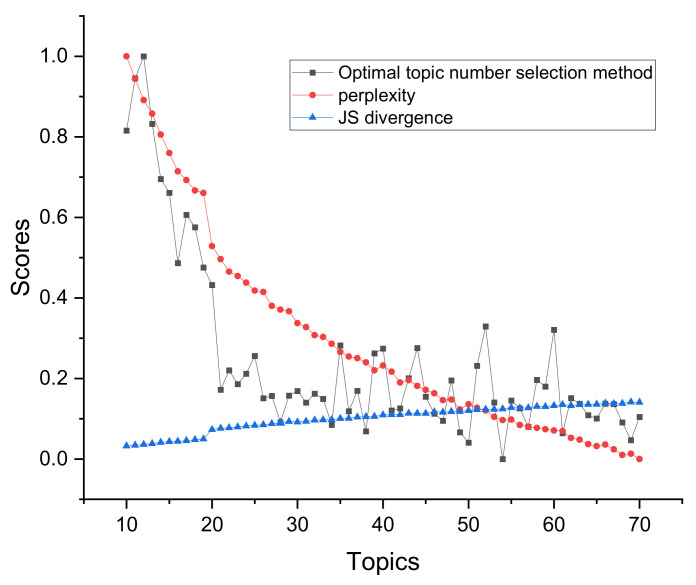
The average topic superiority index.

As can be seen from Figure 35, the optimal number of topics obtained by the perplexity and JS divergence method are both maximum values in the topic number interval.

#### 5.3.2. Comparison of Experimental Results

The results of the optimal number of topics based on isolation, perplexity, and the method proposed in this study are compared as shown in Table 8 and Table 9.

The optimal numbers of topics selected in terms of perplexity and JS divergence are both 70, and the words and probabilities under each topic are shown in Table 9. It can be seen that the higher the number of topics, the more detailed the classification, but certain abnormal topics with particularly low probability will appear. For example, for topic 1 in Table 9, the probability of most topic words is almost 0, which indicates that this theme has almost no possibility to appear in the policy. In fact, it is difficult to get the optimal number of topics based on the perplexity. The optimal value increases with the increase of the number of topics, so it tends to cause the number of selected topics to be too high, and abnormal topics appear. However, the optimal number of topics selected by the model proposed in this study is 54. As can be seen from Table 8, the discrimination of each topic is high with this number of topics, and there are no abnormal topics.

In order to better verify the accuracy of the model proposed in this study, we invited 20 experts to classify the patent policy topics, which were finally divided into five major types of patent creation policies, patent utilization policies, patent protection policies, patent management policies, and patent service policies, including 19 sub-categories and 55 policy topics.

(1) Patent creation policies. The management of patent-creating activities is an important function of the State Intellectual Property Office (SIPO), i.e., the examination and granting of patent rights. The target of the patent creation policy is mainly the creators of patents, including enterprises, research institutes, universities, and individual inventors. There are four main aspects of the patent creation policy: one is to encourage inventions and patent applications through awards and various prizes, the second is to encourage inventions and creations by means of title evaluation or high-tech evaluation, the third is to encourage the declaration of subject projects in key technical fields, and the fourth is the introduction of innovative talents.
(2) Patent utilization policies. Patent utilization is a key step to realize the value of intellectual property rights (IPR). The current patent utilization policy focuses on promoting the industrialization and commercialization of patent achievements. Its policy targets are mainly the creators and users of patents.
(3) Patent protection policies. Starting from 2014, many regional judicial systems in China have established dedicated IP courts for handling IP-related legal disputes. Meanwhile, special IPR actions conducted by the SIPO and local IPR bureaus as the leading units have also made progress in many aspects, strengthening the fight against IPR infringement through administrative protection means and further optimizing the IPR legal, market, and cultural environments. China’s IPR protection policy is currently facing many reforms, including enhancing the statutory means of IPR enforcement, optimizing IPR enforcement procedures, reforming comprehensive IPR administrative enforcement, and broadening IPR dispute resolution channels.
(4) Patent management policies. The patent management policies are public policies that guide and regulate the IP management activities of power holders from the government level. They include upgrading the IP management level of patent rights holders, certification of IP consistent enterprises, management of project and personnel titles, etc. IP management activities are actions exercised by IPR subjects, and the policies are mainly guiding and encouraging policies, whose core objective is to enhance the IP management level of rights holders.
(5) Patent service policies. Patent services are an important way to facilitate a good interface between patent creation, utilization, protection, and management. The content of patent service policies is encapsulated in the links of patent creation, utilization, protection, and management, and it transfers patent services originally provided by rights holders and government departments to third-party institutions in the market. A major shift in China’s current patent policy is to establish and improve the patent service industry system. Typical patent service businesses include public platforms for patent education and operation, patent information and intelligence, patent legal services, patent financial services, patent personnel training, and technology consulting. Promoting the formation of an industrial chain covering the whole process of patent creation, utilization, protection, and management in a competitive market environment is the main policy objective of patent service policy.

The topic contents obtained by the three methods were refined, summarized, and divided into 55 subclasses under five major categories. Then, the accuracy rates under different topics number selection methods were calculated according to the classification results, as shown in Table 10 and Table 11.

Table 10 shows the accuracy of the topic results obtained by each optimal topic number selection method compared with the 55 subclasses obtained by experts. As can be seen from Table 9, the optimal numbers of topics selected by perplexity and JS divergence both reach 70. Although the topics selected by these two methods can cover 52 of the 55 standard topics listed by experts, resulting in the recall reaching 94.55%, there is a higher probability of having invalid topics in the topics selected by this method, which have duplicate connotations or have contents that are difficult to distinguish, specifically including one wrong topic and 17 fuzzy topics that are difficult to classify, thus leading to a decrease in precision and an increase in fuzzy rate. However, based on the topic superiority index proposed in this study, the selected topics have fewer interference topics, higher indicators, and better effect. It is important to note that this study strictly processed all the patent texts, eliminated most of the interfering words and useless words, and combined a large number of synonyms with more important meanings (e.g., combining the names of various banks into banks), so that the final content of the wrong topics was greatly reduced. Without strict treatment of stop words, the presence of a large number of invalid words would lead to an increase in the number of invalid topics obtained in the end. However, as can be seen from Table 10, despite the strict elimination of stop words in this study, invalid topics and some fuzzy topics still appear when the number of topics is too large.

Table 11 shows the results of the experts’ classification of the topics selected under different methods into five major categories of patent policies. As can be seen from Table 9, the 70 topics selected by perplexity and JS divergence can be classified by experts to get the valid five categories, but there are seven topics that cannot be judged, which leads to a high recall, but precision is only 38.46%. Furthermore, all 54 topics selected by the Topic Superiority Index can be reasonably classified into five major categories. However, only 49 topics in the subclasses can be extracted accurately. By comparing the specific topic contents with the 55 topics listed by experts, it is found that the remaining five fuzzy topics cannot be reasonably corresponded under the subclass of technology innovation subsidies in various fields in the patent creation policies. The reason for this is that experts mostly classify the technology innovation subsidies in various fields according to state-supported fields or high-tech fields, such as electronic information, biological and new medicine, aerospace, new materials, high-tech services, new energy and energy conservation, resources and environment, advanced manufacturing and automation, etc. In contrast, the LDA model used in this study yielded topics in which only pharmaceuticals, information, and energy conservation and environmental protection categories could be distinguished. The rest of the categories may be due to fewer policies with relevant classifications or the existence of combined categories in the policies, resulting in some categories being combined into key technologies, high technologies, etc. The specific policy classification of the 54 topics obtained by the model proposed in this study is shown in Table 12.

## 6. Conclusions and Discussion

In this study, we propose the coincidence index based on the perplexity, isolation, and stability, and construct a method for optimal topic number selection of the LDA model. The method is a comprehensive evaluation of the optimal number of topics based on four aspects: the topic model has high predictiveness, there are no duplicate topics among individual topics, the model has repeatability, and each topic has high explanatory power, which is more accurate than the existing methods of judging the optimal number of topics by perplexity or isolation. It can help analysts perform effective and accurate topic extraction in a large amount of text.

To better verify the feasibility of the method proposed in this study, our proposed optimal topic selection method is compared with several commonly used methods using three generic datasets, 20news_groups, WOS_46986, and AG_news, in the empirical part. It is found that our proposed optimal topic selection method has better topics selection results. In addition, we use the real data of the patent policies of each province and city in China (excluding Hong Kong, Macao, and Taiwan) for further verification and analysis. According to the method proposed in this study, 54 topics are finally obtained. Compared with the results of the experts’ classification of patent policies into 5 major categories, 19 sub-categories and 55 subclasses, 49 topics of the 54 topics selected in this study can correspond to the experts’ classification, and the remaining five for the corresponding topics can also match with the sub-categories. Although the topic selection method in this study is applied to the LDA model, the principle can be extended to other topic models.

The method proposed in this study to determine the optimal number of topics for LDA has some practicality; especially when dealing with a large number of texts and a large number of topic classifications, the method has high accuracy and reliability. However, there are still some limitations in this study. First, during the experiment, we found that the deletion of stop words will have an impact on the distribution of words under each topic, and different researchers may have different treatments on the stop words, resulting in subjective bias, which can be improved and studied in the future. Second, some lexical information was not considered in this study for text processing and topic generation, but this information may affect the connotation of the topics, so lexicality and word-associated meanings can also be considered in future studies. Third, due to the natural drawback of the original LDA topic model, it cannot divide well for topics that are too similar. This leads to the fact that even though we were able to pick a relatively optimal number of topics, we did not achieve a result that is consistent with the true number of topics. Therefore, we also validate the use of our proposed method on the word-embedding LDA model, and it turns out that the optimal topic number selection method is also applicable. Since the word-embedding LDA model is better for topic classification, our final selected model is also able to better classify documents by topics. We also found through the literature collection that many works have been done by scholars in improving the accuracy of LDA topic models: for example, the Gaussian LDA model proposed by Das et al. based on the word-embedding technique [32] and the Biterm Topic Model proposed by Pan et al. for sparse matrices such as short texts [33]. Due to time and effort limitations, we do not validate all text classification models in this paper, but since our proposed method is discriminative based on topic features after the text models are trained, it can be applied to these improved topic classification models. We can foresee that if these topic classification models can achieve more accurate text classification results, then the optimal number of topics can also be better selected by using our method, and we will further verify this in our subsequent studies.

## Figures and Tables

**Table 1:** “Topic-word” probability.

Topics	“Topic-Word” Probability	Topic Content
2	Topic1{‘apple’: 0.37895802, ‘pear’: 0.29479387, ‘dog’: 0.21694316, ‘pig’: 0.06072744, ‘cat’: 0.048577495}	Fruits
	Topic2{‘dog’: 0.42677134, ‘cat’: 0.23052387, ‘pig’: 0.21749169, ‘apple’: 0.06457374, ‘pear’: 0.060639422}	Animals
3	Topic1{‘apple’: 0.40487534, ‘pear’: 0.31113106, ‘dog’: 0.21715428, ‘pig’: 0.034434676, ‘cat’: 0.03240472}	Fruits
	Topic2{‘dog’: 0.4494548, ‘cat’: 0.24006312, ‘pig’: 0.23640023, ‘apple’: 0.037318606, ‘pear’: 0.03676326}	Animals
	Topic3{‘dog’: 0.21185784, ‘pig’: 0.2001333, ‘apple’: 0.19875155, ‘pear’: 0.196671, ‘cat’: 0.19258636}	Animals
4	Topic1{‘apple’: 0.3716775, ‘dog’: 0.23012395, ‘pear’: 0.22663262, ‘pig’: 0.08662834, ‘cat’: 0.084937565}	Unknown
	Topic2{‘dog’: 0.4598837, ‘cat’: 0.24295807, ‘pig’: 0.24179693, ‘apple’: 0.02782605, ‘pear’: 0.027535275}	Animals
	Topic3{‘dog’: 0.20344584, ‘apple’: 0.20050451, ‘pig’: 0.20007579, ‘pear’: 0.19842108, ‘cat’: 0.19755279}	Unknown
	Topic4{‘apple’: 0.39690995, ‘pear’: 0.33073652, ‘dog’: 0.2127626, ‘pig’: 0.029939024, ‘cat’: 0.029651856}	Fruits

**Table 2:** “Document-topic” probability of test documents.

Test Documents	Number of Topics = 2	Number of Topics = 3	Number of Topics = 4
Test1	P(Topic1|Test1) = 0.6808042 P(Topic2|Test1) = 0.3191958	P(Topic1|Test1) = 0.59324664 P(Topic2|Test1) = 0.31827474 P(Topic3|Test1) = 0.08847858	P(Topic1|Test1) = 0.06545124 P(Topic2|Test1) = 0.30757654 P(Topic3|Test1) = 0.06323278 P(Topic4|Test1) = 0.56373950
Test2	P(Topic1|Test2) = 0.18206072 P(Topic2|Test2) = 0.81793930	P(Topic1|Test2) = 0.11509656 P(Topic2|Test2) = 0.77113570 P(Topic3|Test2) = 0.11376770	P(Topic1|Test2) = 0.08417302 P(Topic2|Test2) = 0.74760187 P(Topic3|Test2) = 0.08373206 P(Topic4|Test2) = 0.08449302

**Table 3:** Calculated scores of different topic number selection methods.

Topics	Perplexity	JS	Coherence	Stability	Coincidence	Optimal Topic Number Selection Method
2	2.2899	0.0471	0.8536	0.9630	2	100.9598
3	2.2567	0.0601	0.8047	0.9620	3	117.0119
4	2.2526	0.0637	0.7803	0.9623	4	147.0472

**Table 4:** Vocabulary depth and similarity.

d	$R_{1,d}$	$R_{2,d}$	$Jac_{d}$	AJ
1	Results	Technology	0	0
2	Results, Translation	Technology, Contracts	0	0
3	Results, Translation, Technology	Technology, Contracts, Market	0.200	0.067
4	Results, Translation, Technology, Market	Technology, Contracts, Market, Regulations	0.333	0.133
5	Results, Translation, Technology, Market, Contracts	Technology, Contracts, Market, Regulations, Management	0.429	0.193

**Table 5:** The correlation coefficients of the average stability of the model with different number of words.

	sim20	sim50	sim80	sim100	Mean
sim20	-	0.9949	0.9917	0.9901	0.9922
sim50	0.9949	-	0.999	0.9982	0.9974
sim80	0.9917	0.999	-	0.9998	0.996833
sim100	0.9901	0.9982	0.9998	-	0.9960

**Table 6:** Characteristics and differences of data size.

**Dataset**	20news_groups				WOS_46986				AG_news			
**Model**	LDA		word_embedding		LDA		word_embedding		LDA		word_embedding	
**Data size**	100%	80%	100%	80%	100%	80%	100%	80%	100%	80%	100%	80%
**Dictionary length**	10,455	10,455	52,842	52,842	17,465	17,465	37,441	37,441	19,529	19,529	40,461	40,461
**Number of texts**	11314	9051	11314	9051	28,190	22,552	28,190	22,552	120,000	96,000	120,000	96,000
**Bag of Words**	1,006,011	803,520	19,494,540	15,752,120	2,836,169	2,428,971	23,948,780	19,199,000	2,448,795	1,960,293	21,291,360	17,042,060
**Average text length**	88.92	88.78	1723.05	1740.37	100.61	107.71	849.55	851.32	20.41	20.42	177.43	177.52

**Table 7:** The correlation coefficients of the average stability of the model with different numbers of words.

	sim20	sim50	sim80	sim100	Mean
sim20	-	0.9697	0.9362	0.9195	0.9418
sim50	0.9697	-	0.9917	0.9839	0.981767
sim80	0.9362	0.9917	-	0.9986	0.9755
sim100	0.9195	0.9839	0.9986	-	0.967333

**Table 8:** Partial topic terms and probabilities from first five topics when the optimal number of topics is 54 (Topic Superiority Index).

Topic	word1	prob1	word2	prob2	word3	prob3	word4	prob4	word5	prob5
0	enterprise	0.1175	center	0.0885	technology	0.0669	recognition	0.0199	R&D	0.0194
	**word6**	**prob6**	**word7**	**prob7**	**word8**	**prob8**	**word9**	**prob9**	**word10**	**prob10**
	research	0.0191	engineering	0.0177	evaluation	0.0139	technology innovation	0.0135	development	0.0127
1	**word1**	**prob1**	**word2**	**prob2**	**word3**	**prob3**	**word4**	**prob4**	**word5**	**prob5**
	activity	0.1292	propaganda	0.0388	popularization of science	0.0372	organization	0.0296	science	0.0253
	**word6**	**prob6**	**word7**	**prob7**	**word8**	**prob8**	**word9**	**prob9**	**word10**	**prob10**
	innovation	0.0212	hold	0.0201	unit	0.0169	junior	0.0145	science	0.0131
2	**word1**	**prob1**	**word2**	**prob2**	**word3**	**prob3**	**word4**	**prob4**	**word5**	**prob5**
	project	0.0910	capital	0.0774	special funds	0.0316	management	0.0298	use	0.0269
	**word6**	**prob6**	**word7**	**prob7**	**word8**	**prob8**	**word9**	**prob9**	**word10**	**prob10**
	unit	0.0226	method	0.0183	subsidy	0.0165	finance	0.0129	funds	0.0123
3	**word1**	**prob1**	**word2**	**prob2**	**word3**	**prob3**	**word4**	**prob4**	**word5**	**prob5**
	enterprise	0.1126	reward	0.0503	science	0.0315	subsidy	0.0279	exceed	0.0259
	**word6**	**prob6**	**word7**	**prob7**	**word8**	**prob8**	**word9**	**prob9**	**word10**	**prob10**
	support	0.0241	highest	0.0202	capital	0.0195	subsidy	0.0194	disposable	0.0191
4	**word1**	**prob1**	**word2**	**prob2**	**word3**	**prob3**	**word4**	**prob4**	**word5**	**prob5**
	export	0.0786	trade	0.0634	product	0.0447	technology	0.0403	machining	0.0347
	**word6**	**prob6**	**word7**	**prob7**	**word8**	**prob8**	**word9**	**prob9**	**word10**	**prob10**
	enterprise	0.0306	identify	0.0292	international	0.0252	cooperation	0.0203	introduce	0.0181

**Table 9:** Partial topic terms and probabilities from first 10 topics when the optimal number of topics is 70 (perplexity and JS method).

Topic	word1	prob1	word2	prob2	word3	prob3	word4	prob4	word5	prob5
0	enterprise	0.0982	privately operated	0.0893	science	0.0338	institution	0.0300	regulation	0.0228
	**word6**	**prob6**	**word7**	**prob7**	**word8**	**prob8**	**word9**	**prob9**	**word10**	**prob10**
	management	0.0198	private technology	0.0159	science staff	0.0140	operation	0.0136	technology	0.0117
1	**word1**	**prob1**	**word2**	**prob2**	**word3**	**prob3**	**word4**	**prob4**	**word5**	**prob5**
	goal achievement	0.1685	announcement	0.1332	project department	0.0467	industrialization and urbanization	0.0000	expand	0.0000
	**word6**	**prob6**	**word7**	**prob7**	**word8**	**prob8**	**word9**	**prob9**	**word10**	**prob10**
	profession	0.0000	specialization	0.0000	patent	0.0000	patent technology	0.0000	patent application	0.0000
2	**word1**	**prob1**	**word2**	**prob2**	**word3**	**prob3**	**word4**	**prob4**	**word5**	**prob5**
	science	0.0596	innovation	0.0531	technology	0.0260	enterprise	0.0250	autonomy	0.0169
	**word6**	**prob6**	**word7**	**prob7**	**word8**	**prob8**	**word9**	**prob9**	**word10**	**prob10**
	highlights	0.0168	high technology	0.0151	innovation ability	0.0120	research	0.0118	major	0.0115
3	**word1**	**prob1**	**word2**	**prob2**	**word3**	**prob3**	**word4**	**prob4**	**word5**	**prob5**
	handle	0.0339	management	0.0305	request	0.0278	dispute	0.0274	office	0.0183
	**word6**	**prob6**	**word7**	**prob7**	**word8**	**prob8**	**word9**	**prob9**	**word10**	**prob10**
	party	0.0174	complaint	0.0153	secrecy	0.0152	license	0.0143	regulation	0.0139
4	**word1**	**prob1**	**word2**	**prob2**	**word3**	**prob3**	**word4**	**prob4**	**word5**	**prob5**
	patent	0.4377	implementation	0.0343	patent technology	0.0304	institution	0.0278	agent	0.0268
	**word6**	**prob6**	**word7**	**prob7**	**word8**	**prob8**	**word9**	**prob9**	**word10**	**prob10**
	patent application	0.0240	patent right	0.0224	management	0.0209	invention	0.0126	proxy	0.0092

**Table 10:** Comparison of different topics number selection methods (55 subclasses).

Optimal topic Number Selection Method	$T_{extract}$	$T_{correct}$	$T_{standard}$	$T_{wrong}$	Number of Fuzzy Topics	Precision (P)	Recall (R)	F-Value	Fuzzy Rate
JS divergence	70	52	55	1	17	74.29%	94.55%	83.20%	24.29%
Perplexity	70	52	55	1	17	74.29%	94.55%	83.20%	24.29%
Topic superiority index	54	49	55	0	5	90.74%	89.09%	89.91%	9.26%

(Note: *T_extract_* represents the optimal number of topics selected by each method, *T_correct_* represents the number of topics that can determine the subclasses to which they belong, and *T_standard_* represents the number of subclasses predetermined by experts.)

**Table 11:** Comparison of different topics number selection methods (five major categories).

Optimal Topic Number Selection Method	$T_{extract}$	$T_{correct}$	$T_{standard}$	Number of Patent Creation Policies	Number of Patent Utilization Policies	Number of Patent Protection Policies	Number of Patent Management Policies	Number of Patent Service Policies	Precision (P)	Recall (R)	F-Value
JS divergence	13	5	5	16	4	8	18	16	38.46%	100.00%	55.56%
Perplexity	13	5	5	16	4	8	18	16	38.46%	100.00%	55.56%
Topic superiority index	5	5	5	14	4	6	17	13	100.00%	100.00%	100.00%

**Table 12:** Classification of the 54 topics obtained by the model proposed in this study.

Patent Policy Category	Subclass	Topic
Patent creation policies	Reward and subsidy	Topic 3 Technology awards and subsidies; Topic 16 Patent application funding
	Encouraging technology R&D	Topic 9 Encouraging innovation investment; Topic 12 Encouraging technology R&D; Topic 17 Encouraging autonomy innovation
	Subsidies for technological innovation in various fields	Topic 6 Declaration for medical projects; Topic 24 Innovation of agroforestry; Topic 28 High-performance technology creation; Topic 29 Development of key technologies in various fields; Topic 46 Industrial information technology upgrading; Topic 51 Energy-saving and environmental protection projects; Topic 53 Development of high-tech industry
	Competition awards	Topic 35 Publicity of awards for projects of various organizations; Topic 37 Innovation competition
Patent utilization policies	Trade	Topic 4 Import and export trade
	Tax reduction	Topic 7 Tax reduction for the utilization of technical products
	Technology utilization	Topic 23 Technology utilization and promotion
	Achievement transformation	Topic 31 Awards for transformation of scientific and technological achievements
Patent protection policies	Contract guarantee	Topic 14 Technology transfer contract; Topic 22 Technology trade contract
	Infringement dispute	Topic 43 Patent related disputes; Topic 44 Infringement and anti-counterfeiting; Topic 47 IP protection action; Topic 48 Administrative law enforcement
Patent management policies	Special management	Topic 0 Technology identification and evaluation management; Topic 2 Management of the special fund
	Project management	Topic 8 Project acceptance management; Topic 10 Project planning; Topic 32 Project declaration management; Topic 33 Project award management
	Title management	Topic 5 Achievement identification of technical title; Topic 18 Technical personnel qualification audit management
	System management	Topic 21 System reform and management; Topic 27 Economic performance appraisal management; Topic 8 Management of administrative units; Topic 42 System reform and economic performance
	Enterprise management	Topic 19 High-tech declaration management; Topic 25 Standardization management; Topic 30 Improvement of comprehensive capability of IP; Topic 45 Model enterprise of IP management; Topic 49 Technology management of private enterprises
Patent service policies	Science education and talent	Topic 1 Popular science propaganda; Topic 26 Technology services; Topic 34 Education training; Topic 38 Introduction of technical talents
	Service industry	Topic 11 Capacity improvement of service industry; Topic 41 Agent institute; Topic 52 Finance services
	Platform service	Topic 13 Technology transformation platform; Topic 15 Product exhibition; Topic 36 Information platform; Topic 39 Industrial integration platform
	Incubation base	Topic 20 Entrepreneurship and innovation incubation base; Topic 50 Technology development zone

## Data Availability

Not applicable.
